# Jasmonic Acid-Involved *OsEDS1* Signaling in Rice-Bacteria Interactions

**DOI:** 10.1186/s12284-019-0283-0

**Published:** 2019-04-15

**Authors:** Yinggen Ke, Yuanrong Kang, Mengxiao Wu, Hongbo Liu, Shugang Hui, Qinglu Zhang, Xianghua Li, Jinghua Xiao, Shiping Wang

**Affiliations:** National Key Laboratory of Crop Genetic Improvement, National Center of Plant Gene Research (Wuhan), Huazhong Agricultural University, Wuhan, 430070 China

**Keywords:** Enhanced disease susceptibility 1, Bacterial blight, Bacterial leaf streak, Jasmonic acid, Salicylic acid, *Oryza sativa*, *Xanthomonas oryzae*

## Abstract

**Background:**

The function of *Arabidopsis* enhanced disease susceptibility 1 (AtEDS1) and its sequence homologs in other dicots have been extensively studied. However, it is unknown whether rice EDS1 homolog (OsEDS1) plays a role in regulating the rice-pathogen interaction.

**Results:**

In this study, a *OsEDS1*-knouckout mutant (*oseds1*) was characterized and shown to have increased susceptibility to *Xanthomonas oryzae* pv. *oryzae* (*Xoo*) and *Xanthomonas oryzae* pv. *oryzicola* (*Xoc*), suggesting the positive role of *OsEDS1* in regulating rice disease resistance. However, the following evidence suggests that *OsEDS1* shares some differences with *AtEDS1* in its way to regulate the host-pathogen interactions. Firstly, OsEDS1 modulates the rice-bacteria interactions involving in jasmonic acid (JA) signaling pathway, while AtEDS1 regulates *Arabidopsis* disease resistance against biotrophic pathogens depending on salicylic acid (SA) signaling pathway. Secondly, introducing *AtEDS1* could reduce *oseds1* mutant susceptibility to *Xoo* rather than to *Xoc*. Thirdly, exogenous application of JA and SA cannot complement the susceptible phenotype of the *oseds1* mutant, while exogenous application of SA is capable of complementing the susceptible phenotype of the *ateds1* mutant. Finally, *OsEDS1* is not required for *R* gene mediated resistance, while AtEDS1 is required for disease resistance mediated by TIR-NB-LRR class of R proteins.

**Conclusion:**

*OsEDS1* is a positive regulator in rice-pathogen interactions, and shares both similarities and differences with *AtEDS1* in its way to regulate plant-pathogen interactions.

**Electronic supplementary material:**

The online version of this article (10.1186/s12284-019-0283-0) contains supplementary material, which is available to authorized users.

## Background

As sessile organisms plants have evolved sophisticated mechanisms and strategies in responding to biotic and abiotic stimuli and the ever changing environment. Typically, plant immune response to pathogens is initiated by plasma membrane localized pattern recognition receptors (PRRs)-mediated rapid sensing of pathogen-associated molecular patterns (PAMPs) that leads to PAMP-triggered immunity (PTI), or by cytoplasmic resistance (R) proteins-mediated recognition of microbial effectors that activates a strong disease resistance response: effector-triggered immunity (ETI) (Jones and Dangl 2006; Boller and Felix 2009; Zipfel 2009; Thomma et al. 2011). During these immune responses, plant hormones such as salicylic acid (SA) and jasmonic acid (JA) play important roles in mediating various subsets of defense response events (Spoel and Dong 2008). Both synergistic and antagonistic interactions between SA-dependent and JA/ethylene-dependent pathways exist in plant-pathogen interaction (Spoel and Dong 2008).

*Arabidopsis* enhanced disease susceptibility 1 (*AtEDS1*), a pathogen and SA-induced defense responsive gene, encodes a lipase like protein and is required for a set of defense gene expression. It is also required for disease resistance mediated by some Toll–interleukin-1 receptor–nucleotide binding–leucine-rich repeat (TIR-NB-LRR) class of R proteins (Falk et al. 1999; Wiermer et al. 2005). AtEDS1 positively mediates ETI by directly interacting with TIR-NB-LRR type R proteins RPS4 and RPS6 to form the RPS4-AtEDS1 and RPS6-AtEDS1 complexes (Bhattacharjee et al. 2011). The cognate bacterial effectors AvrRps4 and HopA1 interact with AtEDS1 by disrupting RPS4-AtEDS1 and RPS6-AtEDS1 complexes and releasing RPS4 and RPS6, thereby activating ETI and AtEDS1-dependent basal resistance signaling pathway (Bhattacharjee et al. 2011). AtEDS1 functions in SA-dependent pathway by interacting with another lipase like protein, phytoalexin deficient 4 (AtPAD4), to promote SA biosynthesis in a positive feedback manner (Feys et al. 2001). AtEDS1/AtPAD4 mediates the SA-JA/ET signal antagonism as an activator of SA-dependent pathway but a repressor of JA/ET-dependent signaling (Wiermer et al. 2005; Brodersen et al. 2006). AtEDS1/AtPAD4 complexes antagonize JA/ET signal by interacting with MYC2 thereby reducing its binding to target gene promoter (Cui et al. 2018). Simultaneous over-expression of *AtEDS1* and *AtPAD4*, but not individual over-expression of *AtEDS1* or *AtPAD4* leads to autoimmunity and enhanced disease resistance associated with increased SA levels and *PR1* transcripts accumulation (Cui et al. 2017). In addition to *Arabidopsis* AtEDS1, its orthologs from other dicots such as *Nicotiana benthamiana*, *Lycopersicon esculentum*, *Vitis vinifera*, *Glycine max*, *Gossypium barbadense* and *Cicer arietinum* and from monocot *Triticum aestivum* also play positive roles in plant-pathogen interactions, suggesting a conserved role of EDS1 in plant-pathogen interactions (Liu et al. 2002; Peart et al. 2002; Hu et al. 2005; Gao et al. 2010a; Wang et al. 2014; Yan et al. 2016; Chakraborty et al. 2018; Chen et al. 2018). Furthermore, *Lycopersicon esculentum LeEDS1* is required for both TIR-NBS-LRR class resistance (*R*) genes *Bs4* and *N*, and LRR receptor class *R* genes *Ve1* and *Ve2* mediated gene-for-gene resistance (Hu et al. 2005); *Cicer arietinum CaEDS1* is required for coiled-coil (CC)-NBS-LRR class *R* gene *CaRGA* mediated gene-for-gen resistance (Chakraborty et al. 2018). However, it is not clear whether rice EDS1 ortholog, OsEDS1, is also involved in the rice-pathogen interaction.

*Xanthomonas oryzae* pv. *oryzae* (*Xoo*) and *Xanthomonas oryzae* pv. *oryzicola* (*Xoc*) which are two close related pathogens cause rice bacterial blight and rice bacterial leaf streak (Niño-Liu et al. 2006). All two diseases are highly devastating and cause heavy yield losses worldwide. Rice defense-responsive genes are involved in various resistance mechanisms mediated by the JA-dependent pathway, SA-dependent pathway, both JA- and SA-dependent pathways, or both JA- and SA-independent pathway (Qiu et al. 2007; Tao et al. 2009; Fu et al. 2011; Shen et al. 2011; Deng et al. 2012; Ke et al. 2014 and Ke et al. 2017a). In this study, we functionally characterized OsEDS1 for its role in the rice-bacteria interactions by a combination of genetic, molecular, physiological and pathological analyses. These analyses suggest that *OsEDS1* positively regulates rice defense response against *Xoo* and *Xoc* in a JA-dependent manner, which is different from the SA-mediated role of *AtEDS1* in *Arabidopsis* disease resistance.

## Results

### Identification of OsEDS1 and OsPAD4 Protein Interaction

In *Arabidopsis*, AtEDS1 interacts with AtPAD4 and acts as an essential component in the pathogen-induced defense response (Parker et al. 1996; Falk et al. 1999; Feys et al. 2001). To identify the rice EDS1 ortholog, the amino acid sequence of AtEDS1 (accession number: NP_190392) was used to BLAST against the rice genome database. An amino acid sequence (accession number: NP_001063086) encoded by the gene with locus name LOC_Os09g22450 was found and shown to share the highest sequence similarity with AtEDS1. This gene was named as *OsEDS1*, which is a single copy gene in rice genome. OsEDS1 and AtEDS1 share 37% sequence identity and 51% sequence similarity. The most similar regions between OsEDS1 and AtEDS1 are the predicted lipase region that covers approximately 215 amino acids (45% sequence identity and 60% sequence similarity) and harbors the catalytic triad of lipase with the conserved serine (S), aspartate (D), and histidine (H) residues (Additional file 1: Figure S1) (Brady et al. 1990).

**Fig. 1:**
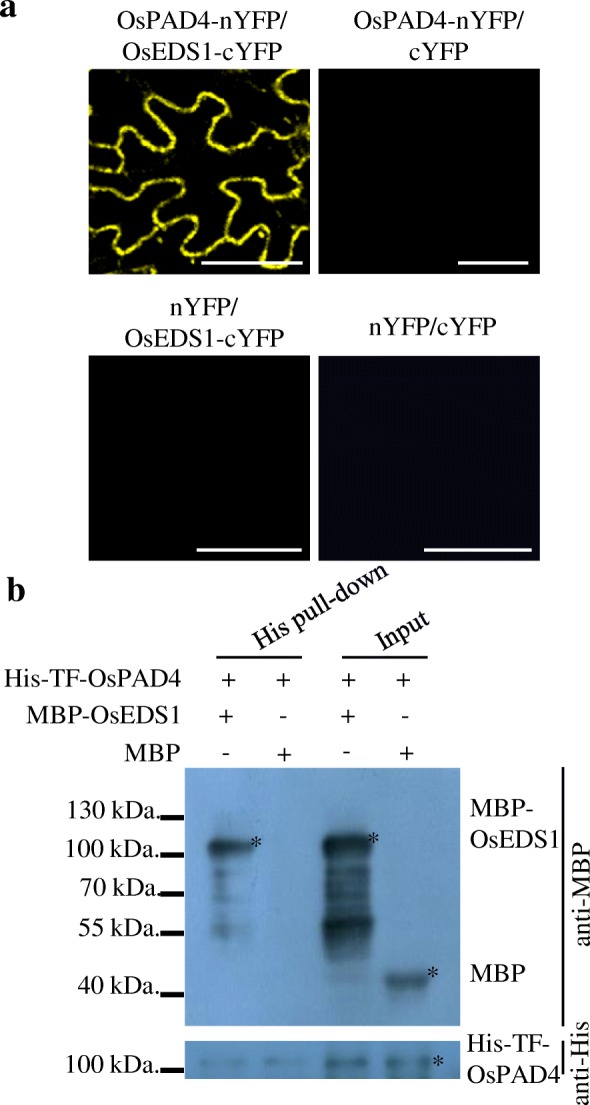
OsPAD4 directly interacted with OsEDS1. **a** OsPAD4 interacts with OsEDS1. BiFC analysis of OsEDS1 and OsPAD4 interaction. Fluorescence can be observed only in tobacco (*Nicotiana benthamiana*) cells co-transfected with OsPAD4-nYFP and OsEDS1-cYFP plasmids. Bars indicate 50 μm. **b** Protein pull-down assay for detection of His-TF-OsPAD4 and MBP-OsEDS1 interaction. Ten micrograms of His-TF-OsPAD4 coupled beads were used to pull down 5 μg MBP or MBP-OsEDS1 protein. Anti-MBP antibody was used to detect output protein. Anti-His antibody was used to detect input protein

To detect whether OsEDS1 directly interacts with the membrane-localized OsPAD4 as the case of AtEDS1 and AtPAD4 in *Arabidopsis*, bimolecular fluorescence complementation (BiFC) assays were performed by transient expression of both OsEDS1-cYFP and OsPAD4-nYFP in plant cells. The YFP signals indicated that OsEDS1 interacted with OsPAD4 at the plasma membrane (Fig. 1a; Additional file 1: Figure S2). OsEDS1 also interacted with *Arabidopsis* AtPAD4 protein in cytoplasm, whereas OsPAD4 interacted with AtEDS1 at the membrane (Additional file 1: Figure S2). In addition, the protein pull-down assay was successfully performed using MBP-OsEDS1 and His-TF-OsPAD4 proteins, demonstrating their interaction *in vitro* (Fig. 1b). These *in vitro* and *in vivo* analyses suggested that OsEDS1 likely interacts with OsPAD4.

### Mutation in *OsEDS1* Influenced Rice Resistance to *X. oryzae*

**Fig. 2:**
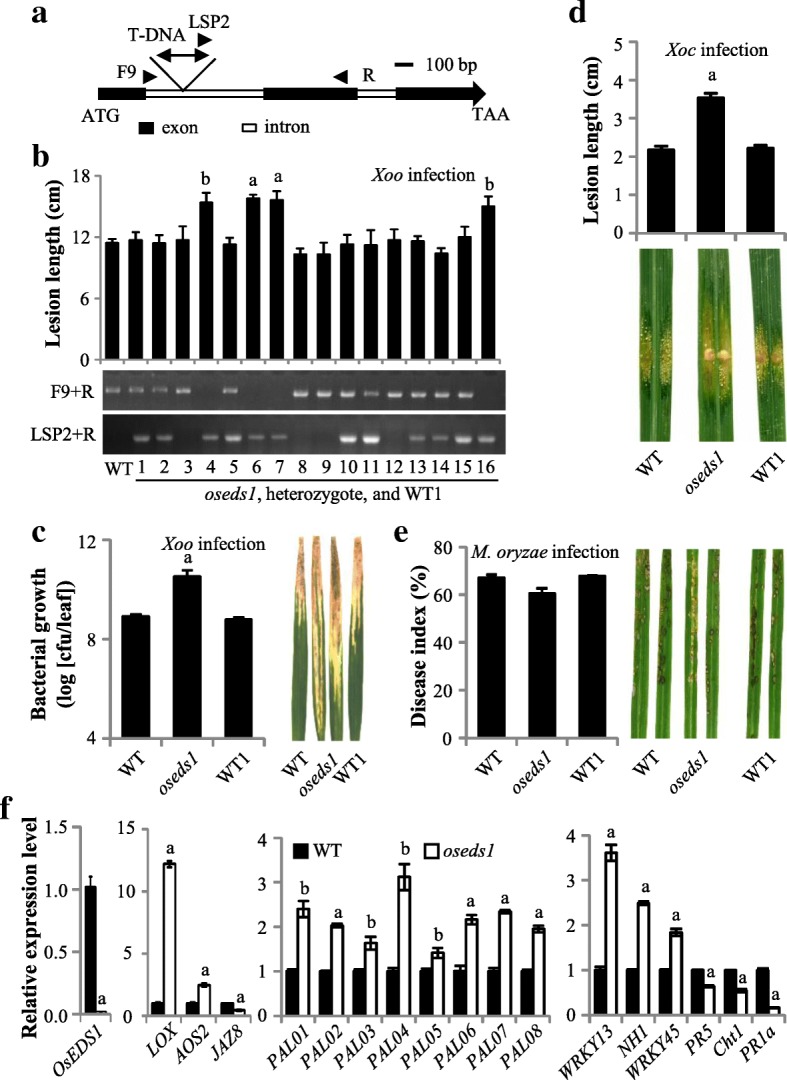
The increased susceptibility of *oseds1* mutant to *X. oryzae* was associated with insertion of T-DNA. Two-tail student’s *t-test*. The “a” and “b” above bar indicate significant differences between wild-type (WT) and *oseds1* plants at *P* < 0.01 and *P* < 0.05, respectively. **a** T-DNA insertion site in *oseds1* mutant. T-DNA was inserted at position 587 of *OsEDS1*, counting the first nucleotide of translation start codon (ATG) as 1. TAA, translation stop codon. Arrows were PCR primers used for examination of the mutant. F9 + R, *OsEDS1* primers; LSP2 + R, T-DNA, and *OsEDS1* primers. **b** Homozygous *oseds1* mutant was more susceptible to *Xoo* than heterozygotes and WT siblings (WT1). Data represent mean ± SE (*n* = 4 to 5). Plants 4, 6, 7 and 16 were homozygous *oseds1* mutants, 1, 2, 5, 10, 11, 13, 14 and 15 were heterozygotes, and 3, 8, 9, and 12 were WT1. **c** Bacterial growth was analyzed at 12 days and disease symptoms at 14 days after *Xoo* infection. Data represent mean ± SE (*n* = 3). **d** *oseds1* was more susceptible to *Xoc*. Lesion length and disease symptom were analyzed at seven days after *Xoc* infection. Data represent mean ± SE (*n* = 10 to 15). **e** *oseds1* had a similar level of susceptibility to *M. oryzae* to that of WT plants. Disease index and disease symptom were analyzed at seven days after *X. oryzae* infection. Data represent mean ± SE (*n* = 23 to 28). **f** *oseds1* affected a set of defense-related genes expression. Data represent mean ± SE (*n* = 3)

We initially isolated *OsEDS1* T-DNA insertion mutant RMD_03Z11KT37 from rice T-DNA insertional library in *Geng* variety Zhonghua 11 (ZH11, susceptible to *Xoo* strain PXO112) (Zhang et al. 2006; Cao et al. 2007). The genomic sequence of RMD_03Z11KT37 was characterized using the gene-specific and T-DNA primers and shown to have a T-DNA insert in the first intron of *OsEDS1* (Fig. 2a). In addition, the mutant progeny with homozygous, heterozygous, or wild-type *OsEDS1* mutation genotypes were obtained by genetic segregation from RMD-03Z11KT37 plants (Additional file 1: Figure S3). These plants showed no obvious phenotypic changes during the developmental stage. The *OsEDS1* transcripts were detected in the WT, insert-negative segregant, and heterozygous *OsEDS1* mutant plants, but not in the homozygous T-DNA mutant (Additional file 1: Figure S3). These results confirm that homozygous RMD_03Z11KT37 is an *OsEDS1*-knockout mutant, which was referred to as *oseds1*.

To determine whether *OsEDS1* is involved in the rice-pathogen interaction, we inoculated *oseds1* mutant with *Xoo* strain PXO112 at the booting (panicle development) stage. The homozygous *oseds1* mutant showed increased susceptibility to *Xoo* compared to heterozygote, WT, and WT sibling plants (Fig. 2b). The growth rate of PXO112 on *oseds1* leaves was significantly higher than that on WT and WT siblings at 12 days after inoculation. The amount of bacteria in *oseds1* was 18.5- and 20.1-fold higher than that in WT and WT sibling plants, respectively (Fig. 2c). Considering the closed relatedness of *Xoo* and *Xoc*, we then inoculated *oseds1* plants with *Xoc* strain RH3 at tillering stage. Results showed that *oseds1* plants were more susceptible to *Xoc* with the lesion length was 3.5 cm for *oseds1* versus 2.2 cm for WT and WT sibling plants (Fig. 2d). Like *Xoo* and *Xoc*, *Magnaporthe oryzae* (*M. oryzae*) is another (hemi) biotrophic pathogen. Previous studies have shown that some genes contribute rice resistance to all these three pathogens, such as *WRKY45* (Tao et al. 2009). To check whether *OsEDS1* play a role in rice-*M. oryzae* interaction, we inoculated *oseds1* mutant with *M. oryzae* isolate Enshi2–2 (N2–2) at one-month-old seedlings. Result showed that *oseds1* mutant had a similar level of susceptibility relative to WT and WT sibling plants (Fig. 2e). These results suggest that *OsEDS1* might act as a positive regulator in rice resistance to *X. oryzae* but not to *M. oryzae*.

To dissect possible defense pathways mediated by *OsEDS1*, we analyzed the expression of a set of rice defense-responsive genes in the *oseds1* mutant. These include *LOX* (lipoxygenase; D14000) and *AOS2* (allene oxide synthase 2; AY062258) involved in JA biosynthesis, *JAZ8* (jasmonate ZIM-domain protein; XP_015612402) related to the JA-dependent signaling pathway (Peng et al. 1994; Mei et al. 2006; Yamada et al. 2012; Ke et al. 2014), *PAL* (phenylalanine ammonia lyase) genes involved in the phenylpropanoid pathway and SA biosynthesis (Duan et al. 2014), *WRKY13*, *NH1* (rice NPR1 homolog 1) and *OsWRKY45* associated with SA-dependent signaling pathway and *Xoo* resistance (Qiu et al. 2007; Tao et al. 2009; Yang et al. 2013), as well as pathogenesis-related protein (PR) genes such as *PR1a* (for acidic pathogenesis-related protein 1; AJ278436), *PR5* (for class 5 pathogenesis-related protein; P28493) and *Cht1* (for chitinase 1; Q42993) (Xiao et al. 2009; Shen et al. 2010; Deng et al. 2012; Ke et al. 2014). The expression levels of *LOX*, *AOS2*, *PAL*s, *WRKY13*, *NH1* and *OsWRKY45* were significantly higher in *oseds1* mutant than those in WT. By contrast, the expression levels of *PR5*, *Cht1*, *PR1a*, and *JAZ8* were significantly lower in *oseds1* mutant than those in WT (Fig. 2f). These results suggest that increased susceptibility of *oseds1* may be associated with impaired JA-related signaling pathways.

### Genetic Validation of OsEDS1’s Role in Rice Resistance

The serine residue at position 143 of OsEDS1 protein and position 123 of AtEDS1 protein is embedded within the GHSSG sequences (Additional file 1: Figure S1) that resemble the GXSXG (X representing any amino acid) motif of eukaryotic lipases catalytic triad (Brady et al. 1990; Wagner et al. 2013). The conserved serine residue of predicted catalytic triad of lipase in OsPAD4 and AtPAD4 is also embedded in the motif similar to GXSXG (Additional file 1: Figure S1). To further verify that the increased susceptibility to *Xoo* and *Xoc* was caused by the mutation of *OsEDS1*, the wild type *OsEDS1* cDNA driven by *OsEDS1* native promoter (*E*^WT^) and mutated *OsEDS1*^S143L^ cDNA driven by *OsEDS1* native promoter (*E*^S143L^) were introduced into the *oseds1* mutant by the *Agrobacterium*-mediated transformation, resulting in 15 independent T_0_ plants for *E*^WT^, and 21 independent T_0_ plants for *E*^S143L^.

**Fig. 3:**
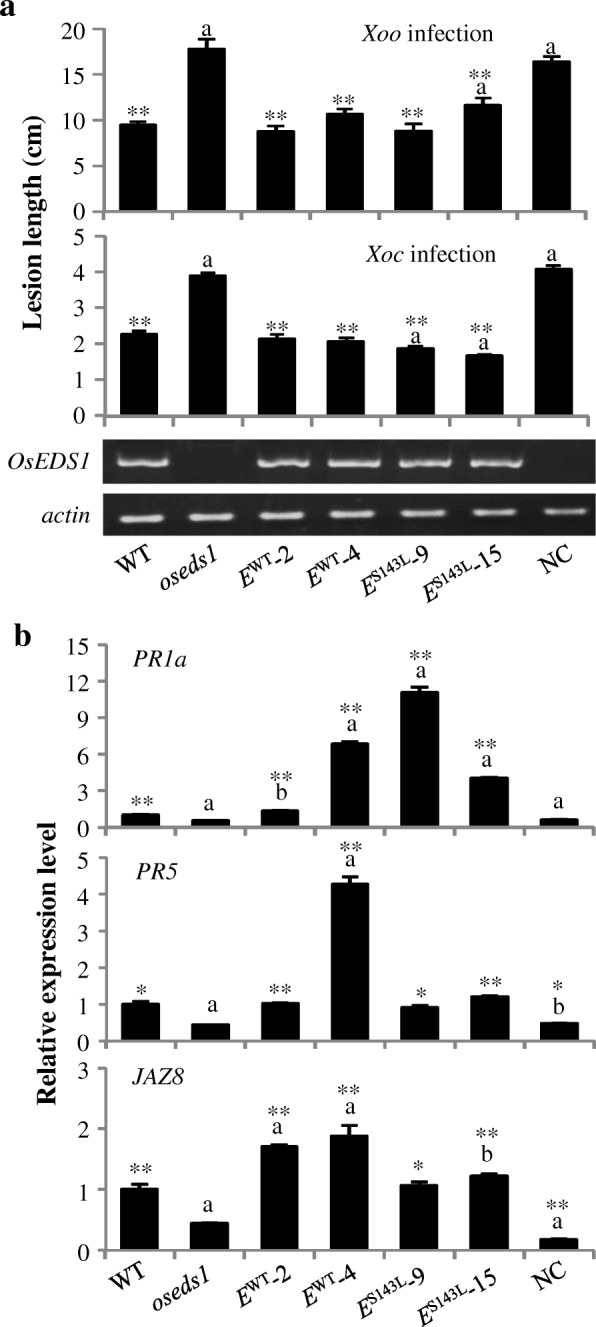
Introduction of *E*^WT^ and *E*^S143L^ constructs complemented *oseds1* mutant phenotype. Two-tail student’s *t-test.* ** and * above bar indicate significant differences compared to *oseds1* at *P* < 0.01 and *P* < 0.05, respectively. The “a” and “b” above bar indicate significant differences compared to wild-type (WT) at *P* < 0.01 and *P* < 0.05, respectively. **a** Introduction of *E*^WT^ and *E*^S143L^ to *oseds1* mutant restored plants showing reduced susceptibility. Data represent mean ± SE (*n* = 10 to 69). **b** Introducing *E*^WT^ and *E*^S143L^ to *oseds1* mutant restored *PR1a*, *PR5*, and *JAZ8* expression. Data represent mean ± SE (*n* = 3)

The T_1_ transgenic plants from five selected T_0_ plants (*E*^WT^-2, *E*^WT^-4, *E*^S143L^-9, *E*^S143L^-15) and empty vector (negative control, NC), were used for further analyses. The *E*^WT^-2, *E*^WT^-4, *E*^S143L^-9, and *E*^S143L^-15 plants showed similar levels of *OsEDS1* expressions to WT (Fig. 3a). These plants were significantly less susceptible to *Xoo* and *Xoc* than that of *oseds1*, and exhibited a similar level of susceptibility as the WT plants (Fig. 3a; Additional file 1: Figure S4a). Consistent with the disease assay, the expression of *PR1a*, *PR5* and *JAZ8* was significantly higher in *E*^WT^ and *E*^S143L^ plants than in *oseds1* and NC plants (Fig. 3b). These results demonstrated that *OsEDS1* was the gene responsible for the mutant phenotype of *oseds1*. However, the conserved S143 residue appeared not required for the *OsEDS1*–mediated rice-*X. oryzae* interactions.

### Partial Complementation of *oseds1* Mutant Phenotype by *AtEDS1*

**Fig. 4:**
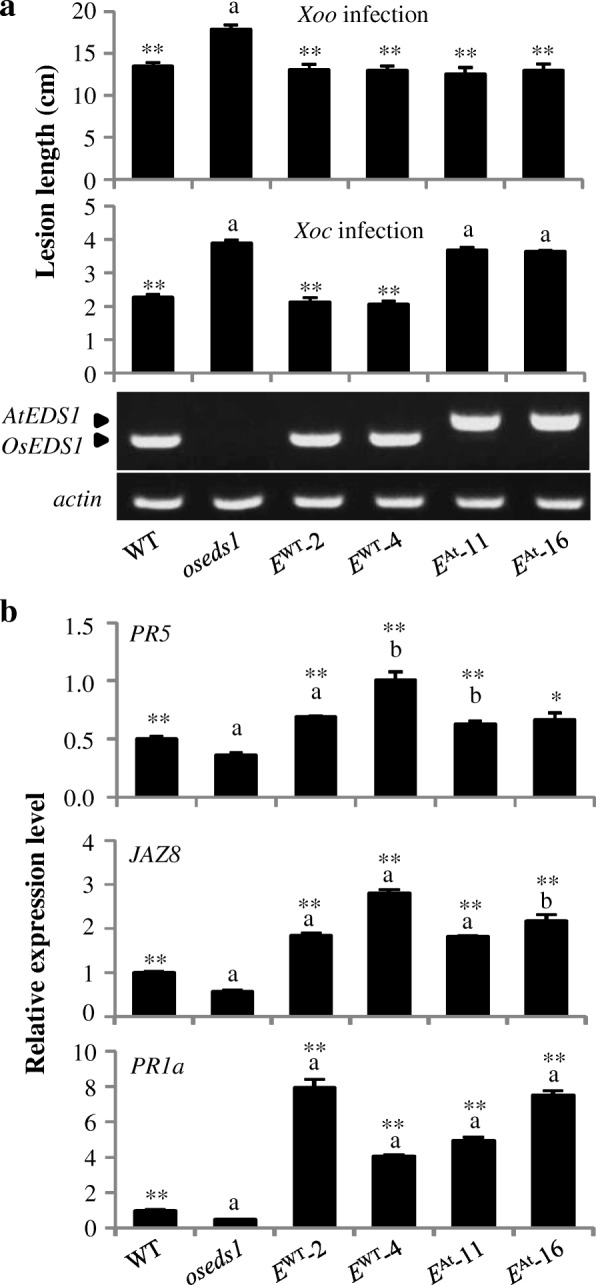
Introduction of *E*^At^ partially complemented *oseds1* mutant phenotype. Data represent mean ± SE (*n* = 10 to 56, and 3 for gene expression). Two-tail student’s *t-test.* ** and * indicate significant differences compared to *oseds1* at *P* < 0.01 and *P* < 0.05, respectively. The “a” and “b” above bar indicate significant differences compared to wild-type (WT) at *P* < 0.01 and *P* < 0.05, respectively. **a** Introducing *E*^At^ to *oseds1* mutant restored plants with partially reduced susceptibility to *Xoo*. **b** Introducing *E*^At^ to *oseds1* mutant restored *PR5*, *JAZ8*, and *PR1a* expression

Rice OsPAD4 functions differently from *Arabidopsis* AtPAD4 in plant-bacteria interactions (Ke et al. 2014). To study if AtEDS1 and OsEDS1 have a similar function in plant-pathogen interactions, the *AtEDS1* cDNA driven by *OsEDS1* native promoter (*E*^At^) was transformed into the *oseds1* mutant. Twenty-six independent transgenic T_0_ plants, *E*^At^-1 to *E*^At^-26, were obtained and verified. The positive plants of *E*^At^-11 and *E*^At^-16 were used for further analyses. The *E*^At^ plants were significantly less susceptible to *Xoo* than that of *oseds1* and exhibited a similar level of susceptibility as the WT plants (Fig. 4a; Additional file 1: Figure S4b). Consistently, the expression levels of *JAZ8*, *PR5* and *PR1a* in *E*^At^ plants were significantly higher than those in *oseds1* mutant (Fig. 4b). However, the *E*^At^ plants had a similar level of susceptibility to *Xoc* relative to *oseds1* mutant (Fig. 4a; Additional file 1: Figure S4b). These results suggest that *AtEDS1* could partially complement *oseds1* mutant phenotype.

### *OsEDS1* is Not Required for LRR Receptor Class *R* Mediated Gene-for-gene Resistance

**Fig. 5:**
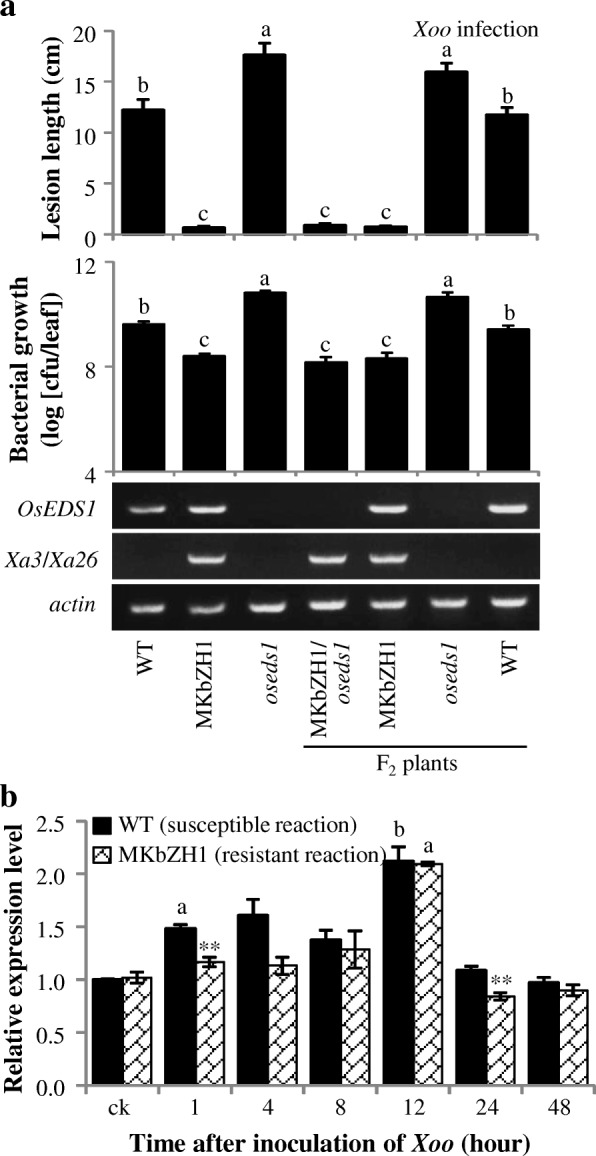
*OsEDS1* was not required for *R*-mediated resistance to *Xoo*. Data represent mean ± SE (*n* = 11 to 15 for lesion length, and 3 for bacterial growth and gene expression). WT, wild-type Zhonghua 11; MKbZH1, transgenic line carrying *R* gene *Xa3/Xa26* with the genetic background of Zhonghua 11. **a** The *oseds1* did not influence *Xa3/Xa26*-mediated resistance. Bacterial growth were analyzed at 12 days after *Xoo* inoculation. Different letters above bars indicate significant differences at *P* < 0.01 by the one-way ANOVA test. **b** *OsEDS1* showed similar expression patterns in both resistant and susceptible reactions. Two-tail student’s *t-test.* The “a” or “b” above bar indicate significant differences between non-inoculated control (ck) plants and inoculated plants at *P* < 0.01 or *P* < 0.05, respectively. The ** above bar indicate significant differences between WT and MKbZH1 plants after the same treatment at *P* < 0.01

In *Arabidopsis*, *AtEDS1* is required for TIR-NBS-LRR class *R* genes *RPP1*, *RPP5* and *RPS4* mediated gene-for-gene resistance (Parker et al. 1996, 2000; Gassmann et al. 1999). In *Lycopersicon esculentum*, *LeEDS1* is required for both TIR-NBS-LRR class *R* genes *Bs4* and *N*, and LRR receptor class *R* genes *Ve1* and *Ve2* mediated gene-for-gene resistance (Hu et al. 2005). There is no TIR-NB-LRR-type *R* gene for resistance to *Xoo* has been identified in rice, and no *R* gene for resistance to *Xoc* has been cloned. To ascertain whether *OsEDS1* plays role in *R* gene-mediated resistance, we crossed transgenic MKbZH1 line with *oseds1* mutant. MKbZH1 is carrying transgenic *R* gene *Xa3/Xa26* with the genetic background of ZH11 (Cao et al. 2007). *Xa3/Xa26*, encoding an LRR receptor kinase-like protein, confers race-specific resistance to *Xoo* including to strain PXO112 (Cao et al. 2007; Gao et al. 2010b; Li et al. 2012). The F_2_ plants (MKbZH1/*oseds1*) carrying *Xa3*/*Xa26* showed similar lesion length and similar bacterial growth with parental plant MKbZH1 (Fig. 5a). The above results were consistent with the similar expression patterns of *OsEDS1* in resistant MKbZH1 line and susceptible ZH11, in general. *OsEDS1* expression level was slightly increased during *Xoo* infection with 12 h having higher expression in both resistant and susceptible reactions, and ZH11 accumulated less *OsEDS1* transcripts at 1 and 24 h (Fig. 5b). These results suggested that *OsEDS1* was not involved in *Xa3/Xa26*–mediated resistance to *Xoo*.

### Role of JA and SA in *OsEDS1*-Mediated Resistance

**Fig. 6:**
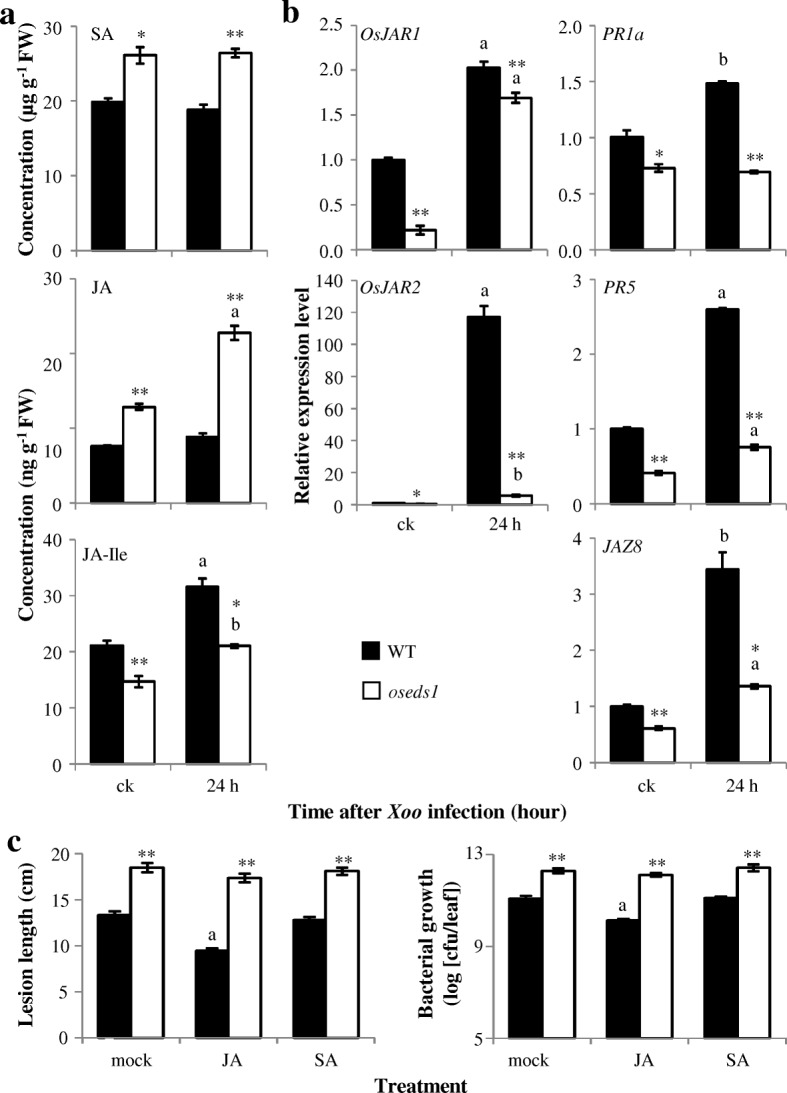
The *oseds1* mutant performance response to *Xoo* inoculation and hormone treatment. Two-tail student’s *t-test.* ** and * above bar indicate significant differences between *oseds1* and wild-type (WT) plants after the same treatment at *P* < 0.01 and *P* < 0.05, respectively. The “a” and “b” above bar indicate significant differences between un-infection (ck) and infection plants at *P* < 0.01 and *P* < 0.05, respectively. FW, fresh weight. **a** Hormone content in *oseds1*. Bars represent mean ± SE (*n* = 3). **b** Genes expression in *oseds1*. Bars represent mean ± SE (*n* = 3). **c** Exogenous application of JA or SA did not reduce the susceptibility of *oseds1* mutant to *Xoo* invasion. Bacterial growth was analyzed at 12 days after *Xoo* infection. Bars represent mean ± SE (*n* = 30 to 38 for lesion length, and 3 for bacterial growth)

In *Arabidopsis*, *AtEDS1* expression can be induced by exogenous SA application. *AtEDS1* expression is also required for pathogen induced SA accumulation and *ateds1* mutant fails to accumulate SA after pathogen infection (Feys et al. 2001). However, *EDS1* homologs expression was not induced by exogenous SA application in monocot plants barley and wheat (Gaudet et al. 2010). Exogenous application of SA did not induce *OsEDS1* expression, but the exogenous application of JA induced *OsEDS1* expression at 1, 3 and 6 h after treatment (Additional file 1: Figure S5). *oseds1* mutant had significantly higher SA content than WT both before and after *Xoo* infection, but *Xoo* infection did not further increase SA content (Fig. 6a; Additional file 1: Figure S6a). The *oseds1* mutant also had significantly higher JA content than WT before and after *Xoo* infection and *Xoo* infection further increased JA content (Fig. 6a; Additional file 1: Figure S6a). However, *oseds1* mutant accumulated less jasmonyl-L-isoleucine (JA-Ile, the most biologically active JA compound) content than WT before and after *Xoo* infection (Fig. 6a). Additionally, the expression levels of two JA-Ile synthases genes *OsJAR1* and *OsJAR2* (Wakuta et al. 2011; Hui et al. 2019) and *PR5*, *PR1a*, and *JAZ8* were induced after *Xoo* inoculation in both WT and *oseds1* mutant plants, with the expression levels of these genes were higher in WT than those in *oseds1* (Fig. 6b; Additional file 1: Figure S6b).

We further analyzed the response of *oseds1* mutant to exogenous application of SA and JA. Exogenous application of JA was shown to induce rice resistance to *Xoo* (Yamada et al. 2012; Ke et al. 2014). Exogenous application of SA may induce rice resistance to *Xoo* in some situation (Song et al. 2001; Xu et al. 2013), but not in other cases (Ke et al. 2014). In WT plants, bacterial blight disease was significantly less severe after JA treatment in comparison with mock treatment, whereas a similar level of the disease was observed in SA- and mock-treated plants (Fig. 6c). However, exogenous application of either JA or SA did not reduce the susceptibility of *oseds1* mutant to *Xoo*, as judged by the similar lesion length and pathogen growth observed in JA- or SA-treated vs. mock-treated *oseds1* plants (Fig. 6c). Nevertheless, exogenous application of JA increased *PR1a* and *JAZ8* gene expression and exogenous application of SA increased *PR1a* and *PR5* gene expression in both *oseds1* and WT plants with the expression levels of these genes being higher in WT than in *oseds1* (Additional file 1: Figure S7). The impaired *PR5* and *PR1a* expression in *oseds1* might be caused by the impaired JA signaling. These results supported that *OsEDS1* may function downstream of JA- biosynthesis in JA-dependent signaling pathway.

## Discussion

EDS1 is a putative triacylglycerol lipase. The positive roles of AtEDS1 and other dicots EDS1s in plant resistance response have been extensively studied (Falk et al. 1999; Liu et al. 2002; Peart et al. 2002; Hu et al. 2005; Wiermer et al. 2005; Gao et al. 2010a; Wang et al. 2014; Yan et al. 2016). The present study showed that rice *oseds1* mutant increased susceptibility to *X. oryzae* compared to WT, suggesting that *OsEDS1* positively regulates rice defense resistance to *X. oryzae*.

Rice OsEDS1 was a sequence ortholog of *Arabidopsis* AtEDS1. OsEDS1 and AtEDS1 interacted with AtPAD4 and OsPAD4, respectively. Both AtEDS1/AtPAD4 and OsEDS1/OsPAD4 mediated plant basal resistance in plant (Fig. 2b-d) (Feys et al. 2001; Ke et al. 2014). *Arabidopsis* AtEDS1 and AtPAD4, as well as rice OsEDS1 and OsPAD4 all contained the GXSXG motif of eukaryotic catalytic triad lipases (Additional file 1: Figure S1; Brady et al. 1990). However, the lipase activity of plant EDS1 and PAD4 has not yet been verified. The predicted lipase catalytic triad residues of EDS1 and PAD4 contain Ser (S), Asp (D), and His (H) in rice and *Arabidopsis* (Additional file 1: Figure S1) (Wagner et al. 2013; Ke et al. 2014; Cui et al. 2018). Mutation of the conversed residue S of GXSXG motif in AtEDS1 or AtPAD4 does not compromise the basal immune response (Louis et al. 2012; Wagner et al. 2013). The predicted lipase regions of OsEDS1 and AtEDS1 have 60% sequence similarity and mutation of the conversed residue S of GXSXG motif in OsEDS1 also did not compromise the basal immune response (Fig. 3a; Additional file 1: Figures S1 and S4). However, the mechanisms underline OsEDS1 and AtEDS1 mediated plant-pathogen interactions may have both similarities and differences. This hypothesis is based on the following evidence.

Firstly, mutation in *OsEDS1* compromised *PR5*, *JAZ8* and *PR1a* expression; introducing *OsEDS1* and *AtEDS1* to *oseds1* mutant, complemented their expression. Secondly, *oseds1* mutant was more susceptible to *Xoo* and *Xoc*; while the introduction of *AtEDS1* reduced the susceptibility of *oseds1* mutant to *Xoo* rather than *Xoc*. Thirdly, *OsEDS1* modulated rice-*X. orzyae* interactions by antagonistically affecting SA-JA-related signaling pathway, as a repressor of SA-dependent and an activator of JA-dependent, and exogenous application of SA and JA cannot complement susceptible phenotype of *oseds1* mutant; while AtEDS1 regulates *Arabidopsis*-pathogen interaction by antagonistically affecting SA–ET/JA interaction, as an activator of SA-dependent and a repressor of ET/JA-dependent, and exogenous application of SA can complement susceptible phenotype of *ateds1* mutant. (Falk et al. 1999; Wiermer et al. 2005; Brodersen et al. 2006; Cui et al. 2018). Fourthly, OsEDS1 physically interacted with OsPAD4 which mediates the rice-bacteria interactions via the JA signaling pathway (Ke et al. 2014), while AtEDS1 associates with AtPAD4 which enhances *Arabidopsis* resistance to pathogens by activating SA signaling pathway and suppressing ET/JA-dependent pathway (Cui et al. 2017). Finally, there is no TIR-NB-LRR-type *R* gene for resistance to *Xoo* has been identified in monocots, and no *R* gene for resistance to *Xoc* has been cloned in rice. *OsEDS1* was not required for LRR receptor class *R* gene *Xa3/Xa26* mediated resistance to *Xoo* (Cao et al. 2007; Liu et al. 2018), while AtEDS1 is required for disease resistance mediated by TIR-NB-LRR class of R proteins (Falk et al. 1999; Wiermer et al. 2005).

Although the closed relatedness of *Xoo* and *Xoc* which belong to the same species and are both biotrophic pathogens, they have different pathogenic mechanisms. *Xoo* invades rice plants through hydathodes or wounds and multiplies in the intercellular spaces then enter into and spread through the xylem. *Xoc* penetrates the leaves of rice plants through stomata and wounds and multiplies in the substomatal cavity and then colonizes the intercellular spaces of the parenchyma and mesophyll cells (Niño-Liu et al. 2006). Os*WRKY45–2* and *OsMPK6* promote rice resistance to both *Xoo* and *Xoc* (Tao et al. 2009; Shen et al. 2010). OsMPK3 which plays a negative role in the rice defense against *Xoo* was not involved in rice resistance to *Xoc* infection (Xiong and Yang 2003; Ma et al. 2017). Our present study showed that *oseds1* was more susceptible to *Xoo* and *Xoc*, and introducing *AtEDS1* could reduce *oseds1* susceptibility to *Xoo*, but not to *Xoc*. Thus, the mechanisms underline rice defense against *Xoo* and *Xoc* may have both similarities and differences.

JA originates from α-linolenic acid of chloroplast membranes by oxidative processes (Wasternack and Song 2017; Wasternack and Strnad 2018). Consequently, JA perception takes only upon formation of JA-Ile which is catalyzed by JA-Ile synthase (Fonseca et al. 2009). Following JA-Ile sensed by the co-receptor COI1 (coronatine insensitive 1)/JAZ, the JAZ repressor is de-repressed via COI1-mediated ubiquitination and 26S proteasomal degradation which results in releasing of the transcription factors and binding to the promoters of JA-responsive genes. Plant defense response requires intact JA signaling (Kazan and Manners 2013). The analysis of the endogenous level of JA, the expression of JA responsive genes and the sensitivity to exogenous JA clearly showed that mutation in *OsEDS1* impaired JA-Ile synthetic enzyme genes *OsJAR1*/*2* expression resulting in less JA-Ile accumulation downstream of JA biosynthesis in JA-related signaling pathway. In *Arabidopsis*, *atjar1* and *atmyc2* mutants reduce sensitivity to JA and activate SA biosynthesis (Nickstadt et al. 2004; Laurie-Berry et al. 2006). In *oseds1* mutant, the activated SA biosynthesis and SA signaling may cause by reduced JA-Ile level. Constitutively high level of SA may alter the sensitivity of downstream signaling components to SA (Chen et al. 1997). In *Arabidopsis*, defense against biotrophic pathogens is SA-dependent, while defense against necrotrophic pathogens is JA-dependent (Robert-Seilaniantz et al. 2011). In rice, benzothiadiazole (SA analog) and JA can induce resistance against both biotrophic and necrotrophic pathogens (De Vleesschauwer et al. 2013). Clearly, the defense model of defense hormones SA and JA in rice is strikingly different form that in *Arabidopsis*.

## Conclusion

Like *AtEDS1*, *OsEDS1* can positively regulate rice disease resistance. *OsEDS1* and AtEDS1 have both similarities and differences in their way to regulate plant-pathogen interactions. However, the mechanism underlying *OsEDS1* regulated SA- and JA- related signaling pathway could not be exhaustively addressed in the present work. Thus, further studies are needed to focus on resolving the mechanism of *OsEDS1*-mediated defense in rice may provide further insight into this perspective.

## Methods

### Bioinformatics Analysis

The amino acid sequence of *Arabidopsis* AtEDS1 (accession number of protein database in National Center for Biotechnology Information [NCBI; http://www.ncbi.nlm.nih.gov]: NP_190392) was used to identify its ortholog from rice genome database (http://blast.ncbi.nlm.nih.gov/Blast) by the BLASTP program (Altschul et al. 1997). The rice amino acid sequence NP_001063086 (gene locus LOC_Os09g22450) showed the highest sequence identity to AtEDS1. The genomic sequence of LOC_Os09g22450 was used to search Knowledge-based Oryza Molecular biological Encyclopedia database (http://cdna01.dna.affrc.go.jp/cDNA/Wblast2.html), and a full-length cDNA AK100117 (cDNA clone J023007E18) corresponding to LOC_Os09g22450 from rice variety Nipponbare (*Oryza sativa ssp. geng*) was identified. The rice *EDS1* cDNA clone was kindly provided by RIKEN Yokohama Institute (Suzuki et al. 1997).

### *In vivo* and *in vitro* Protein Interaction Assays

Biomolecular fluorescence complementation (BiFC) assays were applied to study the interactions of OsEDS1 and OsPAD4 as well as their interaction with *Arabidopsis* AtEDS1 or AtPAD4 based on the previously reported procedure (Yuan et al. 2010). Open reading frames encoding OsEDS1 and OsPAD4 were obtained by PCR amplification from cDNA clones J023007E18 and J100075 L24 using the gene specific primers (Additional file 2: Table S1) and cloned into the vectors encoding pS1301-N-terminal region of yellow fluorescence protein (nYFP) or pS1301-C-terminal region of YFP (cYFP) to generate OsPAD4-nYFP and OsEDS1-cYFP constructs, respectively. Full length cDNAs encoding AtEDS1 and AtPAD4 were obtained by PCR amplification using *Arabidopsis* Col-0 variety leaf cDNA and gene specific primers (Additional file 2: Table S1) and cloned into the pS1301-nYFP or pS1301-cYFP vectors to generate AtPAD4-nYFP and AtEDS1-cYFP constructs, respectively. The constructs were co-transformed into rice (*O. sativa* ssp. *geng*) variety Zhonghua 11 stem protoplasts (Yuan et al. 2010). For BiFC assays in tobacco (*Nicotiana benthamiana*), OsPAD4-nYFP, OsEDS1-cYFP, pS1301-nYFP and pS1301-cYFP plasmids were transformed into tobacco plants via *A. tumefaciens* strain GV3101-pM90. The fluorescence was observed by confocal microscopy (TCS SP2; Leica).

For the protein pull-down assay, full-length cDNAs of *OsEDS1* and *OsPAD4* were cloned into the pMAL or pCOLD vectors, respectively, and transformed into *Escherichia coli* strain BL21 to express maltose binding protein (MBP)-tag OsEDS1 (MBP-OsEDS1) and His-TF-tag OsPAD4 (His-TF-OsPAD4). Total soluble protein (10 μg) containing His-TF-OsPAD4 was incubated with Ni Sepharose™ 6 Fast Flow (17–5318-01, GE Healthcare) at 4 °C for 1 h, and then the beads were treated with 5% skimmed milk for 3 h at 4 °C to block them. The beads were washed 5 times and 5 μg MBP-OsEDS1 was added. The incubation continued for 3 h and the beads were washed 5 times. The beads were boiled in 1× SDS loading buffer and separated by 10% SDS-PAGE. The anti-MBP antibody (E8032L, NEB) was used to detect the MBP-tagged protein. Anti-His antibody (AE003, ABclonal) was used to detect input protein.

### Gene Constructs and Rice Transformation

For the complementation test of *oseds1* mutant, *OsEDS1* and *AtEDS1* full-length cDNAs were placed behind a 2-kb *OsEDS1* putative promoter, which was amplified from Zhonghua 11 genomic DNA (Additional file 2: Table S1). These constructs were referred as *E*^WT^ and *E*^At^, respectively. The OsEDS1 harbors the catalytic triad of lipase with the conserved serine, aspartate, and histidine residues (Brady et al. 1990). We substituted the conserved 143 serine (S) residue of OsEDS1 with leucine (L) using gene-specific primers (Additional file 2: Table S1), and place this mutated OsEDS1 gene behind the *OsEDS1* putative promoter. The resulting construct was referred as *E*^S143L^. These three constructs were made using the pCAMBIA2301 vector. Subsequently, the constructs were individually introduced into *Agrobacterium tumefaciens* strain EHA105 by electroporation. The *Agrobacterium*-mediated rice transformation was performed using calli derived from the embryos of *oseds1* mutant (Lin and Zhang 2005).

### Pathogen Inoculation

To evaluate bacterial blight disease, rice plants were inoculated with Philippine *Xoo* strain PXO112 using the leaf-clipping method at the booting stage (Chen et al. 2002; Ke et al. 2017b). Disease was scored by measuring lesion length at about 14 days after inoculation. The bacterial growth rate in rice leaves was measured by counting the colony forming units (Sun et al. 2004; Ke et al. 2017b).

To evaluate bacterial leaf streak disease, rice plant at tillering stage were inoculated with Chinese *Xoc* strain RH3 by the penetration method using a syringe (Ke et al. 2014). Disease was scored by measuring lesion length at about seven days after inoculation.

To evaluate rice blast disease, one-month-old seedlings were inoculated with Chinese *M. oryzae* isolate Enshi2–2 (N2–2) by spraying method (Chen et al. 2003). Disease was scored according to Cheng et al. (2015).

### Hormone Treatment

Rice plants growing in greenhouse until the 6-leaf stage were sprayed with 250 μM JA, 500 μM SA or solution (mock spray) containing 0.1% (*v*/v) methanol and 0.015% (v/v) Tween 20 until uniformly wet. The treated plants were kept in sealed plastic shade for two days and then inoculated with *Xoo* strain PXO112.

### Gene Expression Analysis and Quantification of SA and JA

Healthy rice leaves or leaf segments next to bacterial infection sites were used for RNA isolation and phytohormone quantification. Quantitative reverse transcription (qRT)-PCR was conducted using gene-specific primers (Additional file 2: Table S2) as described previously (Qiu et al. 2007). The expression level of the rice actin gene was used to standardize the RNA sample amount for each qRT-PCR. The expression level relative to control was presented.

The same samples used for gene expression analysis were used for phytohormone quantification. JA and SA were quantified using an ultrafast liquid chromatograph/electrospray ionization/tandem mass spectrometry system as described previously (Liu et al. 2012).

### Statistical Analyses

The statistical significance of differences between control and sample treatments were assessed using the pair-wise *t*-test installed in the Microsoft Office Excel program. The multiple samples were analyzed by one-way ANOVA using Tukey’s multiple comparison test in software R (The R project for Statistical Computing; https://www.r-project.org) as described by Deng et al. (2018).

## Additional Files

Additional file 1: **Figure S1.** Alignment of the lipase domains of rice OsEDS1 and OsPAD4 as well as *Arabidopsis* AtEDS1 and AtPAD4. **Figure S2.** PAD4s interacts with EDS1s. **Figure S3.** Analysis of T-DNA insertion mutant RMD_03Z11KT37. **Figure S4.** *oseds1* mutant complementation assays. *oseds1* defense to *Xoo* but not *Xoc*. **Figure S5.** Effect of exogenous SA or JA application on *OsEDS1* expression in rice plants. **Figure S6.** The *oseds1* mutant shows higher SA and JA contents than those of wild-type (WT) plant. **Figure S7.** The *oseds1* mutant had lower expression levels of *PR1a*, *PR5* and *JAZ8* genes than WT before and after JA or SA treatment. (DOC 73 kb)

Additional file 2: **Table S1.** PCR primers used for construction of vectors, detection of positive transgenic plants, mutant analysis, and sequencing. **Table S2.** Primers used for quantitative PCR in gene expression analysis. (PPT 3837 kb)

## Abbreviations

AOS2: Allene Oxide Synthase 2
Cht1: Chitinase 1
EDS1: Enhanced Disease Susceptibility 1
JA: Jasmonic acid
JAZ: Jasmonate ZIM-domain protein
LOX: Lipoxygenase
*M. oryzae*: *Magnaporthe oryzae*
PAD4: Phytoalexin Deficient 4
*PAL*: Phenylalanine Ammonia Lyase
PR: Pathogenesis-Related Protein
SA: Salicylic Acid
*Xoc*: *Xanthomonas oryzae* pv. *oryzicola*
*Xoo*: *Xanthomonas oryzae* pv. *oryzae*

## References

[CR1] Altschul SF, Madden TL, Schäffer AA, Zhang J, Zhang Z, Miller W, Lipman DJ (1997). Gapped BLAST and PSI-BLAST: a new generation of protein database search programs. Nucleic Acids Res.

[CR2] Bhattacharjee S, Halane MK, Kim SH, Gassmann W (2011). Pathogen effectors target *Arabidopsis* EDS1 and alter its interactions with immune regulators. Science.

[CR3] Boller T, Felix G (2009). A renaissance of elicitors: perception of microbe-associated molecular patterns and danger signals by pattern-recognition receptors. Annu Rev Plant Biol.

[CR4] Brady L, Brzozowski AM, Derewenda ZS, Dodson E, Dodson G, Tolley S, Turkenburg JP, Christiansen L, Huge-Jensen B, Norskov L (1990). A serine protease triad forms the catalytic centre of a triacylglycerol lipase. Nature.

[CR5] Brodersen P, Petersen M, Bjørn Nielsen H, Zhu S, Newman MA, Shokat KM, Rietz S, Parker J, Mundy J (2006). *Arabidopsis* MAP kinase 4 regulates salicylic acid- and jasmonic acid/ethylene-dependent responses via EDS1 and PAD4. Plant J.

[CR6] Cao Y, Ding X, Cai M, Zhao J, Lin Y, Li X, Xu C, Wang S (2007). Expression pattern of a rice disease resistance gene *Xa3*/*Xa26* is differentially regulated by the genetic backgrounds and developmental stages that influence its function. Genetics.

[CR7] Chakraborty J, Priya P, Dastidar SG, Das S (2018). Physical interaction between nuclear accumulated CC-NB-ARC-LRR protein and WRKY64 promotes EDS1 dependent *Fusarium* wilt resistance in chickpea. Plant Sci.

[CR8] Chen G, Wei B, Li G, Gong C, Fan R, Zhang X (2018). *TaEDS1* genes positively regulate resistance to powdery mildew in wheat. Plant Mol Biol.

[CR9] Chen H, Wang S, Xing Y, Xu C, Hayes PM, Zhang Q (2003). Comparative analyses of genomic locations and race specificities of loci for quantitative resistance to *Pyricularia grisea* in rice and barley. Proc Natl Acad Sci U S A.

[CR10] Chen H, Wang S, Zhang Q (2002). New gene for bacterial blight resistance in rice located on chromosome 12 identified from Minghui 63, an elite restorer line. Phytopathology.

[CR11] Chen Z, Iyer S, Caplan A, Klessig DF, Fan B (1997). Differential accumulation of salicylic acid and salicylic acid-sensitive catalase in different rice tissues. Plant Physiol.

[CR12] Cheng H, Liu H, Deng Y, Xiao J, Li X, Wang S (2015). The WRKY45-2 WRKY13 WRKY42 transcriptional regulatory cascade is required for rice resistance to fungal pathogen. Plant Physiol.

[CR13] Cui H, Gobbato E, Kracher B, Qiu J, Bautor J, Parker JE (2017). A core function of EDS1 with PAD4 is to protect the salicylic acid defense sector in *Arabidopsis* immunity. New Phytol.

[CR14] Cui H, Qiu J, Zhou Y, Bhandari DD, Zhao C, Bautor J, Parker JE (2018). Antagonism of transcription factor MYC2 by EDS1/PAD4 complexes bolsters salicylic acid defense in *Arabidopsis* effector-triggered immunity. Mol Plant.

[CR15] De Vleesschauwer D, Gheysen G, Höfte M (2013). Hormone defense networking in rice: tales from a different world. Trends Plant Sci.

[CR16] Deng H, Liu H, Li X, Xiao J, Wang S (2012). A CCCH-type zinc finger nucleic acid-binding protein quantitatively confers resistance against rice bacterial blight disease. Plant Physiol.

[CR17] Deng Y, Liu H, Zhou Y, Zhang Q, Li X, Wang S (2018). Exploring the mechanism and efficient use of a durable gene-mediated resistance to bacterial blight disease in rice. Mol Breed.

[CR18] Duan L, Liu H, Li X, Xiao J, Wang S (2014). Multiple phytohormones and phytoalexins are involved in disease resistance to *Magnaporthe oryzae* invaded from roots in rice. Physiol Plant.

[CR19] Falk A, Feys BJ, Frost LN, Jones JD, Daniels MJ, Parker JE (1999). EDS1, an essential component of *R* gene-mediated disease resistance in *Arabidopsis* has homology to eukaryotic lipases. Proc Natl Acad Sci U S A.

[CR20] Feys BJ, Moisan LJ, Newman MA, Parker JE (2001). Direct interaction between the *Arabidopsis* disease resistance signaling proteins, EDS1 and PAD4. EMBO J.

[CR21] Fonseca S, Chini A, Hamberg M, Adie B, Porzel A, Kramell R, Miersch O, Wasternack C, Solano R (2009). (+)-7-iso-Jasmonoyl-L-isoleucine is the endogenous bioactive jasmonate. Nat Chem Biol.

[CR22] Fu J, Liu H, Li Y, Yu H, Li X, Xiao J, Wang S (2011). Manipulating broad-spectrum disease resistance by suppressing pathogen-induced auxin accumulation in rice. Plant Physiol.

[CR23] Gao F, Shu X, Ali MB, Howard S, Li N, Winterhagen P, Qiu W, Gassmann W (2010). A functional EDS1 ortholog is differentially regulated in powdery mildew resistant and susceptible grapevines and complements an *Arabidopsis eds1* mutant. Planta.

[CR24] Gao J, Zhao J, Xu C, Li X, Wang S (2010). Development of rice germplasms conferring high-level and broad-spectrum resistance to *Xanthomonas oryzae* pv. *oryzae* at both seedling and adult stages. Mol. Plant Breed.

[CR25] Gassmann W, Hinsch ME, Staskawicz BJ (1999). The *Arabidopsis RPS4* bacterial-resistance gene is a member of the TIR-NBS-LRR family of disease-resistance genes. Plant J.

[CR26] Gaudet DA, Wang Y, Penniket C, Lu ZX, Bakkeren G, Laroche A (2010). Morphological and molecular analyses of host and nonhost interactions involving barley and wheat and the covered smut pathogen *Ustilago hordei*. Mol Plant-Microbe Interact.

[CR27] Hu G, deHart AK, Li Y, Ustach C, Handley V, Navarre R, Hwang CF, Aegerter BJ, Williamson VM, Baker B (2005). EDS1 in tomato is required for resistance mediated by TIR-class *R* genes and the receptor-like *R* gene *Ve*. Plant J.

[CR28] Hui S, Hao M, Liu H, Xiao J, Li X, Yuan M, Wang S (2019). The group I GH3 family genes encoding JA-Ile synthetase act as positive regulator in the resistance of rice to *Xanthomonas oryzae* pv. *oryzae*. Biochem Biophys Res Commun.

[CR29] Jones JD, Dangl JL (2006). The plant immune system. Nature.

[CR30] Kazan K, Manners JM (2013). MYC2: the master in action. Mol Plant.

[CR31] Ke Y, Deng H, Wang S (2017). Advances in understanding broad-spectrum resistance to pathogens in rice. Plant J.

[CR32] Ke Y, Hui S, Yuan M (2017b) *Xanthomonas oryzae* pv. *oryzae* inoculation and growth rate on Rice by leaf clipping method. Bio-Protoc 7. 10.21769/BioProtoc.2568 10.21769/BioProtoc.2568 PMC8438489 34595251

[CR33] Ke Y, Liu H, Li X, Xiao J, Wang S (2014). Rice *OsPAD4* functions differently from Arabidopsis *AtPAD4* in host-pathogen interactions. Plant J.

[CR34] Laurie-Berry N, Joardar V, Street IH, Kunkel BN (2006). The *Arabidopsis thaliana JASMONATE INSENSITIVE 1* gene is required for suppression of salicylic acid-dependent defenses during infection by *Pseudomonas syringae*. Mol Plant-Microbe Interact.

[CR35] Li HJ, Li XH, Xiao JH, Wing RA, Wang SP (2012). Ortholog alleles at *Xa3/Xa26* locus confer conserved race-specific resistance against *Xanthomonas oryzae* in rice. Mol Plant.

[CR36] Lin Y, Zhang Q (2005). Optimising the tissue culture conditions for high efficiency transformation of *indica* rice. Plant Cell Rep.

[CR37] Liu H, Li X, Xiao J, Wang S (2012). A convenient method for simultaneous quantification of multiple phytohormones and metabolites: application in study of rice-bacterium interaction. Plant Methods.

[CR38] Liu Y, Cao Y, Zhang Q, Li X, Wang S (2018). A cytosolic triosephosphate isomerase is a key component in XA3/XA26-mediated resistance. Plant Physiol.

[CR39] Liu Y, Schiff M, Marathe R, Dinesh-Kumar SP (2002). Tobacco *Rar1*, *EDS1* and *NPR1/NIM1* like genes are required for N-mediated resistance to tobacco mosaic virus. Plant J.

[CR40] Louis J, Gobbato E, Mondal HA, Feys BJ, Parker JE, Shah J (2012). Discrimination of Arabidopsis PAD4 activities in defense against green peach aphid and pathogens. Plant Physiol.

[CR41] Ma H, Chen J, Zhang Z, Ma L, Yang Z, Zhang Q, Li X, Xiao J, Wang S (2017). MAPK kinase 10.2 promotes disease resistance and drought tolerance by activating different MAPKs in rice. Plant J.

[CR42] Mei C, Qi M, Sheng G, Yang Y (2006). Inducible overexpression of a rice allene oxide synthase gene increases the endogenous jasmonic acid level, PR gene expression, and host resistance to fungal infection. Mol Plant-Microbe Interact.

[CR43] Nickstadt A, Thomma BP, Feussner I, Kangasjärvi J, Zeier J, Loeffler C, Scheel D, Berger S (2004). The jasmonate-insensitive mutant *jin1* shows increased resistance to biotrophic as well as necrotrophic pathogens. Mol Plant Pathol.

[CR44] Niño-Liu DO, Ronald PC, Bogdanove AJ (2006). *Xanthomonas oryzae* pathovars: model pathogens of a model crop. Mol Plant Pathol.

[CR45] Parker JE, Feys BJ, van der Biezen EA, Noël L, Aarts N, Austin MJ, Botella MA, Frost LN, Daniels MJ, Jones JD (2000). Unravelling *R* gene-mediated disease resistance pathways in *Arabidopsis*. Mol Plant Pathol.

[CR46] Parker JE, Holub EB, Frost LN, Falk A, Gunn ND, Daniels MJ (1996). Characterization of *edsl*, a mutation in *Arabidopsis* suppressing resistance to *Peronospora parasíiíca* specified by severa1 different RPP genes. Plant Cell.

[CR47] Peart JR, Cook G, Feys BJ, Parker JE, Baulcombe DC (2002). An EDS1 orthologue is required for N-mediated resistance against tobacco mosaic virus. Plant J.

[CR48] Peng YL, Shirano Y, Ohta H, Hibino T, Tanaka K, Shibata D (1994). A novel lipoxygenase from rice: primary structure and specific expression upon incompatible infection with rice blast fungus. J Biol Chem.

[CR49] Qiu D, Xiao J, Ding X, Xiong M, Cai M, Cao Y, Li X, Xu C, Wang S (2007). *OsWRKY13* mediates rice disease resistance by regulating defense-related genes in salicylate- and jasmonate-dependent signaling. Mol Plant-Microbe Interact.

[CR50] Robert-Seilaniantz A, Grant M, Jones JD (2011). Hormone crosstalk in plant disease and defense: more than just jasmonate-salicylate antagonism. Annu Rev Phytopathol.

[CR51] Shen X, Liu H, Yuan B, Li X, Xu C, Wang S (2011). OsEDR1 negatively regulates rice bacterial resistance via activation of ethylene biosynthesis. Plant Cell Environ.

[CR52] Shen X, Yuan B, Liu H, Li X, Xu C, Wang S (2010). Opposite functions of a rice mitogen-activated protein kinase during the process of resistance against *Xanthomonas oryzae*. Plant J.

[CR53] Song FM, Ge XC, Zheng Z, Xie Y (2001). Benzothiadiazole-induced systemic acquired resistance in rice against *Xanthomonas oryzae* pv. *oryzae*. Chin J Rice Sci.

[CR54] Spoel SH, Dong X (2008). Making sense of hormone crosstalk during plant immune responses. Cell Host Microbe.

[CR55] Sun X, Cao Y, Yang Z, Xu C, Li X, Wang S, Zhang Q (2004). *Xa26*, a gene conferring resistance to *Xanthomonas oryzae* pv. *oryzae* in rice, encodes an LRR receptor kinase-like protein. Plant J.

[CR56] Suzuki Y, Yoshitomo-Nakagawa K, Maruyama K, Suyama A, Sugano S (1997). Construction and characterization of a full length-enriched and a 5′-end-enriched cDNA library. Gene.

[CR57] Tao Z, Liu H, Qiu D, Zhou Y, Li X, Xu C, Wang S (2009). A pair of allelic WRKY genes play opposite role in rice-bacteria interactions. Plant Physiol.

[CR58] Thomma BP, Nurnberger T, Joosten MH (2011). Of PAMPs and effectors: the blurred PTI-ETI dichotomy. Plant Cell.

[CR59] Wagner S, Stuttmann J, Rietz S, Guerois R, Brunstein E, Bautor J, Niefind K, Parker JE (2013). Structural basis for signaling by exclusive EDS1 heteromeric complexes with SAG101 or PAD4 in plant innate immunity. Cell Host Microbe.

[CR60] Wakuta S, Suzuki E, Saburi W, Matsuura H, Nabeta K, Imai R, Matsui H (2011). OsJAR1 and OsJAR2 are jasmonyl-L-isoleucine synthases involved in wound- and pathogen-induced jasmonic acid signalling. Biochem Biophys Res Commun.

[CR61] Wang J, Shine MB, Gao QM, Navarre D, Jiang W, Liu C, Chen Q, Hu G, Kachroo A (2014). Enhanced disease susceptibility1 mediates pathogen resistance and virulence function of a bacterial effector in soybean. Plant Physiol.

[CR62] Wasternack C, Song S (2017). Jasmonates: biosynthesis, metabolism, and signaling by proteins activating and repressing transcription. J Exp Bot.

[CR63] Wasternack C, Strnad M (2018). Jasmonates: news on occurrence, biosynthesis, metabolism and action of an ancient group of signaling compounds. Int J Mol Sci.

[CR64] Wiermer M, Feys BJ, Parker JE (2005). Plant immunity: the EDS1 regulatory node. Curr Opin Plant Biol.

[CR65] Xiao W, Liu H, Li Y, Li X, Xu C, Long M, Wang S (2009). A rice gene of de novo origin negatively regulates pathogen-induced defense response. PLoS One.

[CR66] Xiong L, Yang Y (2003). Disease resistance and abiotic stress tolerance in rice are inversely modulated by an abscisic acid-inducible mitogen-activated protein kinase. Plant Cell.

[CR67] Xu J, Audenaert K, Hofte M, De Vleesschauwer D (2013). Abscisic acid promotes susceptibility to the rice leaf blight pathogen *Xanthomonas oryzae* pv. *oryzae* by suppressing salicylic acid-mediated defenses. PLoS One.

[CR68] Yamada S, Kano A, Tamaoki D, Miyamoto A, Shishido H, Miyoshi S, Taniguchi S, Akimitsu K, Gomi K (2012). Involvement of OsJAZ8 in jasmonate-induced resistance to bacterial blight in rice. Plant Cell Physiol.

[CR69] Yan Z, Xingfen W, Wei R, Jun Y, Zhiying M (2016). Island cotton enhanced disease susceptibility 1 gene encoding a lipase-like protein plays a crucial role in response to *Verticillium dahliae* by regulating the SA level and H_2_O_2_ accumulation. Front Plant Sci.

[CR70] Yang D, Yang Y, He Z (2013). Roles of plant hormones and their interplay in Rice immunity. Mol Plant.

[CR71] Yuan M, Chu Z, Li X, Xu C, Wang S (2010). The bacterial pathogen *Xanthomonas oryzae* overcomes rice defenses by regulating host copper redistribution. Plant Cell.

[CR72] Zhang J, Li C, Wu C, Xiong L, Chen G, Zhang Q, Wang S (2006). RMD: a rice mutant database for functional analysis of the rice genome. Nucleic Acids Res.

[CR73] Zipfel C (2009). Early molecular events in PAMP-triggered immunity. Curr Opin Plant Biol.

