# Supplementary material for: Jasmonic Acid-Involved OsEDS1 Signaling in Rice-Bacteria Interactions
Source: Rice (N Y). 2019 Apr 15;12:25. doi: 10.1186/s12284-019-0283-0 (PMC6465387; doi:10.1186/s12284-019-0283-0)
Supplement: Supplementary file 1 — Figure S1. Alignment of the lipase domains of rice OsEDS1 and OsPAD4 as well as Arabidopsis AtEDS1 and AtPAD4. Figure S2. PAD4s interacts with EDS1s. Figure S3. Analysis of T-DNA insertion mutant RMD_03Z11KT37. Figure S4. oseds1 mutant complementation assays. oseds1 defense to Xoo but not Xoc. Figure S5. Effect of exogenous SA or JA application on OsEDS1 expression in rice plants. Figure S6. The oseds1 mutant shows higher SA and JA contents than those of wild-type (WT) plant. Figure S7. The oseds1 mutant had lower expression levels of PR1a, PR5 and JAZ8 genes than WT before and after JA or SA treatment. (DOC 73 kb) [file 12284_2019_283_MOESM1_ESM.doc]

**Table S1.** PCR primers used for construction of vectors, detection of positive transgenic plants, mutant analysis, and sequencing

| Gene (accession number) | Primer name | Forward primer (5’-3’) | Reverse primer (5’-3’) | Product size (nt) | Use |
| --- | --- | --- | --- | --- | --- |
| *OsEDS1*  (AK100117) | *OsEDS1*BiFC-F/R | TGGCGCGCCACTAGTGGATCC^a^ATGCCGGCGGCGGCGGCGCTG | CATCCCGGGAGCGGTACC^a^CCAGGGCACAAGTTTCGCGAT | 1863 | BiFC vector construction |
|  | *OsEDS1*pMAL-F/R | GGAAGGATTTCAGAATTC^a^ATGCCGGCGGCGGCGGCG CTG | GTCGACTCTAGAGGATCC^a^TTACCAGGGCACAAGTTTCGCGAT | 1866 | pMAL vector construction |
|  | *OsEDS1*-F9/R | GCTTTGCTGTTTCGCTACCT | TCCCAAGTAATCCACGCAAAC | 1639 | Mutant analysis |
|  | *OsEDS1*-PROF2/R2 | CCCGAATTC^d^gccagatcctgggggagtac | ACGCGTCGAC^e^CGTCGGGGAGCGGGAGCGCG | 2000 | OsEDS1 promoter amplification |
|  | *OsEDS1*-F10/R5 | GCGTCGAC ^e^GT ATG CCG GCG GCG GCG GCG CTG | GGGGTACC^f^ CCAGG GCACA AGTTT CGCGA | 1866 | *E*^WT^ vector construction |
|  | *OsEDS1*-F14/R14 | TATTCACAGGCCATTTATCA | TATTGAACCCCCTGATAAAT |  | Mutate 143S to 143L |
| *OsPAD4*  (AK243523) | *OsPAD4*BiFC-F/R | TGGCGCGCCACTAGTGGATCC^a^ATGCTTCTTCTTCGTCGTCGT | CATCCCGGGAGCGGTACC^a^CCTTCTCTCCCGGCCATGGGT | 1648 | BiFC vectors construction |
|  | *OsPAD4*-F11/R12 | CGGGATCC^b^ATGCTTCTTCTTCGTCGTCGT | GCTCTAGA^c^CTACCTTCTCTCCCGGCCAT | 1651 | pCOLD vectors construction |
| *AtEDS1*  (NM_114678) | *AtEDS1*-F3/R2 | TGGCGCGCCACTAGTGGATCC^a^ATGGCGTTTGAAGCTCTTAC | CATCCCGGGAGCGGTACC^a^GGTATCTGTTATTTCATCCA | 1869 | BiFC vectors construction |
|  | *AtEDS1*-F1/R1 | GCGTCGAC^e^atggcgtttgaagctcttac | GGGGTACC^f^TCAGGTATCTGTTATTTCAT | 1869 | *E*^AT^ vector construction |
| *AtPAD4*  (NM_115103) | *AtPAD4*-F1/R1 | TGGCGCGCCACTAGTGGATCC^a^ATGGACGATTGTCGATTCGAGA | CATCCCGGGAGCGGTACC^a^AGTCTCCATTGCGTCACTCTCA | 1623 | BiFC vectors construction |
| Vector | LSP2 | GAAGTACTCGCCGATAGTGGAAACC |  |  | Mutant analysis |

^a^The underlined nucleotides are homologous recombination arms.

^b^The underlined nucleotides are the digestion site of *Bam*HI.

^c^The underlined nucleotides are the digestion site of *Xba*I.

^d^The underlined nucleotides are the digestion site of *Eco*RI.

^e^The underlined nucleotides are the digestion site of *Sal*I.

^f^The underlined nucleotides are the digestion site of *Kpn*I.

**Table S2. Primers used for quantitative PCR in gene expression analysis**

| Gene (accession number) | Primer name | Forward primer (5’-3’) | Reverse primer (5’-3’) | Product size (nt) |
| --- | --- | --- | --- | --- |
| *OsEDS1*  (AK100117) | *OsEDS1-*F/ R | TCAGTTGGATCCCCAGCAA | TCCCAAGTAATCCACGCAAAC | 120 |
| *PR1a*  (AJ278436) | *PR1a*-F/R | CGTCTTCATCACCTGCAACTACTC | CATGCATAAACACGTAGCATAGC | 130 |
| *Actin*  (X15865) | *Actin*-F/R | TGTATGCCAGTGGTCGTACCA | CCAGCAAGGTCGAGACGAA | 121 |
| *PAL01*  (AK058306) | *PAL1*-F/R | AGGAGCTCGGCTGCGTATT | ATGCCGAGGAACACCTTGTT | 120 |
| *PAL02*  (AK100346) | *PAL2*-F/R | AGCTGGTCAACGAGTTCTACA  ACA | GAGGGAGTTGACGTCCTGGTT | 123 |
| *PAL03*  (AK240729) | *PAL3*-F/R | AGCACCACCCTGGACAGATC | CTGTCCTGCCTCAGCTTCGT | 120 |
| *PAL04*  (Os05g35290) | *PAL4*-F/R | ATCGACCTGCGCCACATC | GAGTTGGTGCTCAGCGTCTTC | 120 |
| *PAL05*  (Os02g41670) | *PAL5*-F/R | ATCCAAGGTGGCTTCTTCGA | GGCAAGGACAGCAAGAATGTTC | 121 |
| *PAL06*  (AK068993) | *PAL6*-F/R | GGGCAACCCAGTGACCAA | CGATTGCCTCGTCGGTCTT | 110 |
| *PAL07*  (AK067801) | *PAL7*-F/R | CCAACCCTGTGACCAACCAT | GATCAAGAACGTCGAGGACATG | 120 |
| *PAL08*  (AK061959) | *PAL8*-F/R | GCTTCTTCGAGTTGCAGCCTAA | CAGGACCTCGGCGAGGAT | 120 |
| *PR5*  (X68197) | *PR5*-F/R | CAACAGCAACTACCAAGTCGTCTT | CAAGGTGTCGTTTTATTCATCAACTTT | 120 |
| *AOS2*  (AY062258) | *AOS2*-F/R | CAATACGTGTACTGGTCGAATGG | AAGGTGTCGTACCGGAGGAA | 120 |
| *WRKY13*  (EF143611) | *WRKY13*  -F/R | TCAGTGGAGAAGCGGGTGGTG | GGGTGGTTGTGCTCGAAGGAG | 253 |
| NH1  (AY923983) | NH1-F/R | CACGCCTAAGCCTCGGATTA | TCAGTGAGCAGCATCCTGACTAG | 125 |
| *WRKY45*  ([GQ331932](http://www.plantphysiol.org/lookup/external-ref?link_type=GEN&access_num=GQ331932&atom=%2Fplantphysiol%2F151%2F2%2F936.atom)) | *WRKY45*  -F/R | TTCCTTGTTGATG TGTCGTCTCA | CCCCCAGCTCATA ATCAAGAAC | 131 |
| *LOX*  (D14000) | *LOX*-F/R | GCATCCCCAACAGCACATC | AATAAAGATTTGGGAGTGACATATTGG | 110 |
| *JAZ8*  (AK108738) | *JAZ8*-F/R | GAAGGCTCAACAGCTGACCAT | TTGGTGGACGGGAAGTTCTC | 120 |
