# Supplementary material for: Jasmonic Acid-Involved OsEDS1 Signaling in Rice-Bacteria Interactions
Source: Rice (N Y). 2019 Apr 15;12:25. doi: 10.1186/s12284-019-0283-0 (PMC6465387; doi:10.1186/s12284-019-0283-0)
Supplement: Supplementary file 2 — Table S1. PCR primers used for construction of vectors, detection of positive transgenic plants, mutant analysis, and sequencing. Table S2. Primers used for quantitative PCR in gene expression analysis. (PPT 3837 kb) [file 12284_2019_283_MOESM2_ESM.ppt]

## Slide 1
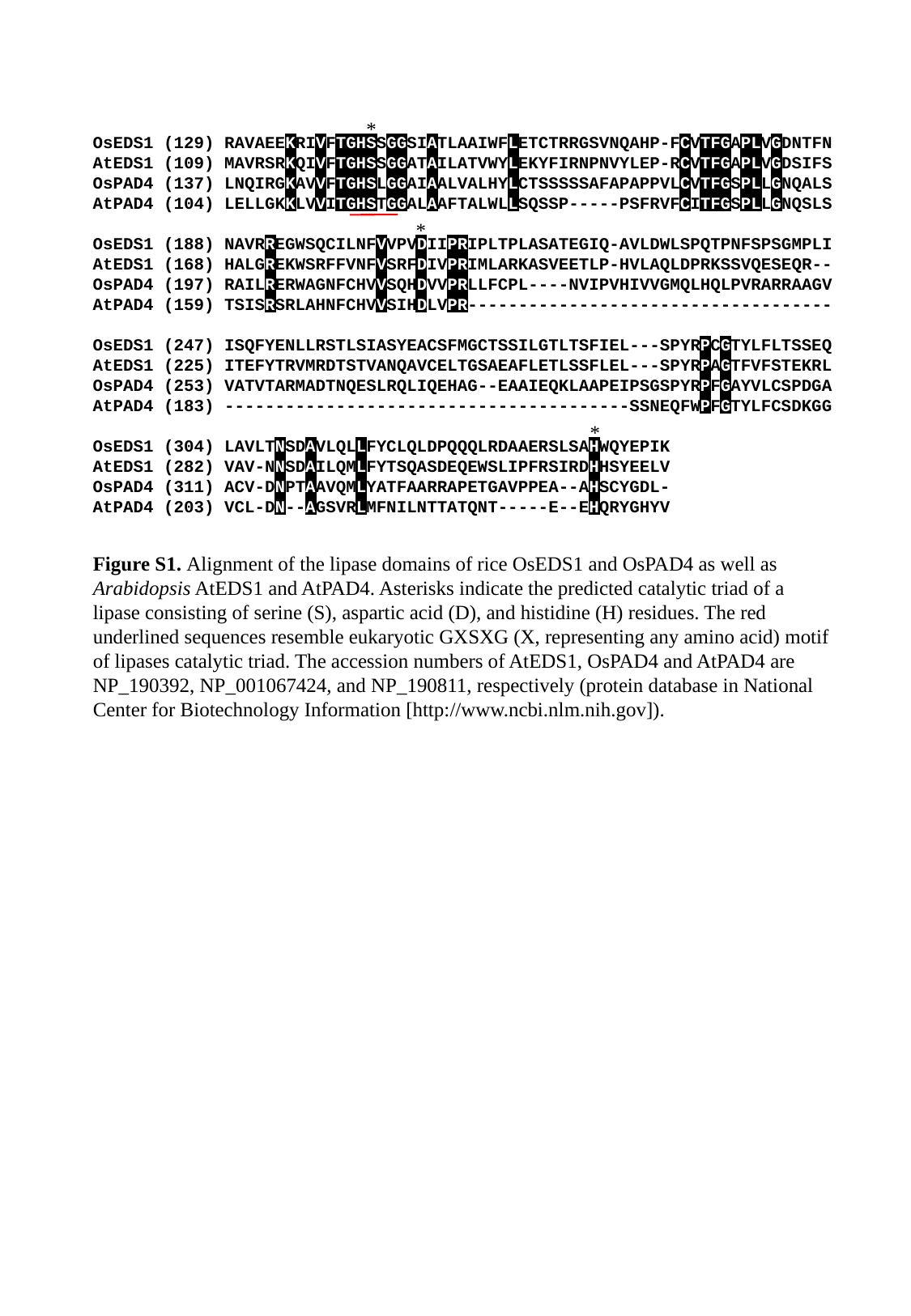

*
OsEDS1 (129) RAVAEEKRIVFTGHSSGGSIATLAAIWFLETCTRRGSVNQAHP-FCVTFGAPLVGDNTFN
AtEDS1 (109) MAVRSRKQIVFTGHSSGGATAILATVWYLEKYFIRNPNVYLEP-RCVTFGAPLVGDSIFS
OsPAD4 (137) LNQIRGKAVVFTGHSLGGAIAALVALHYLCTSSSSSAFAPAPPVLCVTFGSPLLGNQALS
AtPAD4 (104) LELLGKKLVVITGHSTGGALAAFTALWLLSQSSP-----PSFRVFCITFGSPLLGNQSLS
OsEDS1 (188) NAVRREGWSQCILNFVVPVDIIPRIPLTPLASATEGIQ-AVLDWLSPQTPNFSPSGMPLI
AtEDS1 (168) HALGREKWSRFFVNFVSRFDIVPRIMLARKASVEETLP-HVLAQLDPRKSSVQESEQR--
OsPAD4 (197) RAILRERWAGNFCHVVSQHDVVPRLLFCPL----NVIPVHIVVGMQLHQLPVRARRAAGV
AtPAD4 (159) TSISRSRLAHNFCHVVSIHDLVPR------------------------------------
OsEDS1 (247) ISQFYENLLRSTLSIASYEACSFMGCTSSILGTLTSFIEL---SPYRPCGTYLFLTSSEQ
AtEDS1 (225) ITEFYTRVMRDTSTVANQAVCELTGSAEAFLETLSSFLEL---SPYRPAGTFVFSTEKRL
OsPAD4 (253) VATVTARMADTNQESLRQLIQEHAG--EAAIEQKLAAPEIPSGSPYRPFGAYVLCSPDGA
AtPAD4 (183) ----------------------------------------SSNEQFWPFGTYLFCSDKGG
OsEDS1 (304) LAVLTNSDAVLQLLFYCLQLDPQQQLRDAAERSLSAHWQYEPIK
AtEDS1 (282) VAV-NNSDAILQMLFYTSQASDEQEWSLIPFRSIRDHHSYEELV
OsPAD4 (311) ACV-DNPTAAVQMLYATFAARRAPETGAVPPEA--AHSCYGDL-
AtPAD4 (203) VCL-DN--AGSVRLMFNILNTTATQNT-----E--EHQRYGHYV
*
*
Figure S1. Alignment of the lipase domains of rice OsEDS1 and OsPAD4 as well as Arabidopsis AtEDS1 and AtPAD4. Asterisks indicate the predicted catalytic triad of a lipase consisting of serine (S), aspartic acid (D), and histidine (H) residues. The red underlined sequences resemble eukaryotic GXSXG (X, representing any amino acid) motif of lipases catalytic triad. The accession numbers of AtEDS1, OsPAD4 and AtPAD4 are NP_190392, NP_001067424, and NP_190811, respectively (protein database in National Center for Biotechnology Information [http://www.ncbi.nlm.nih.gov]).

## Slide 2
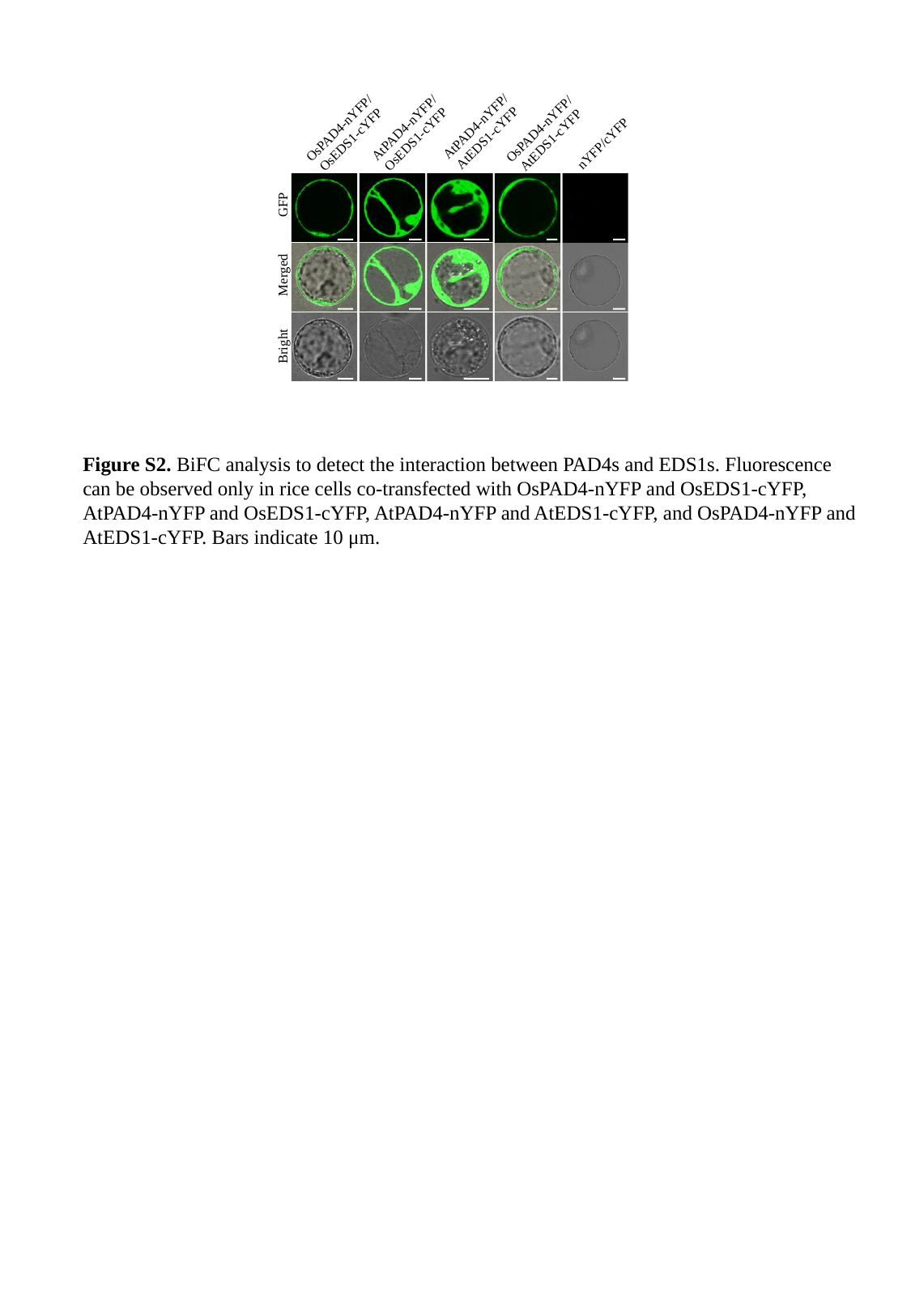

AtPAD4-nYFP/
OsEDS1-cYFP
OsPAD4-nYFP/
OsEDS1-cYFP
OsPAD4-nYFP/
AtEDS1-cYFP
nYFP/cYFP
GFP
Merged
Bright
AtPAD4-nYFP/
AtEDS1-cYFP
Figure S2. BiFC analysis to detect the interaction between PAD4s and EDS1s. Fluorescence can be observed only in rice cells co-transfected with OsPAD4-nYFP and OsEDS1-cYFP, AtPAD4-nYFP and OsEDS1-cYFP, AtPAD4-nYFP and AtEDS1-cYFP, and OsPAD4-nYFP and AtEDS1-cYFP. Bars indicate 10 μm.

## Slide 3
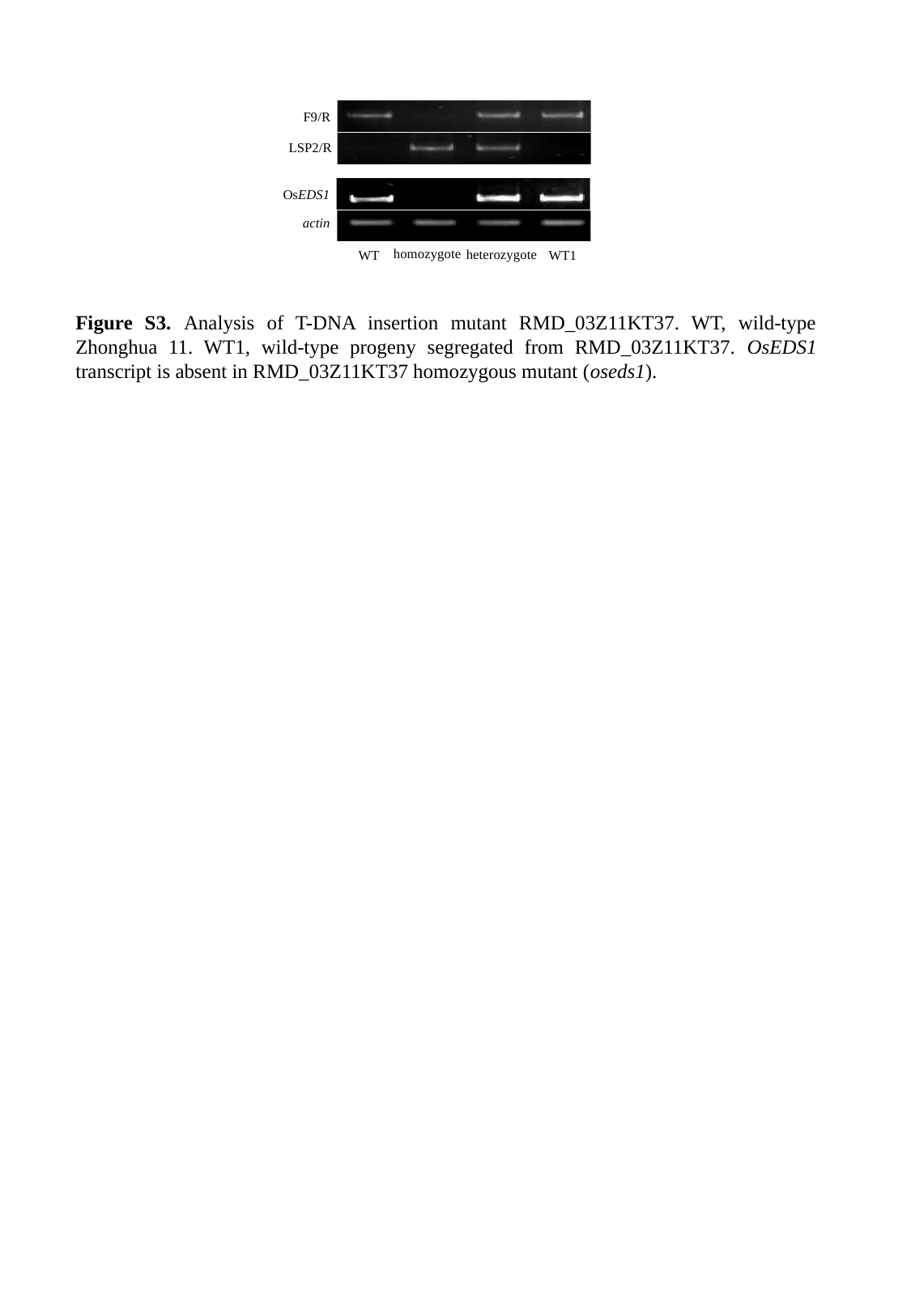

F9/R
LSP2/R
OsEDS1
actin
homozygote
heterozygote
WT
WT1
Figure S3. Analysis of T-DNA insertion mutant RMD_03Z11KT37. WT, wild-type Zhonghua 11. WT1, wild-type progeny segregated from RMD_03Z11KT37. OsEDS1 transcript is absent in RMD_03Z11KT37 homozygous mutant (oseds1).

## Slide 4
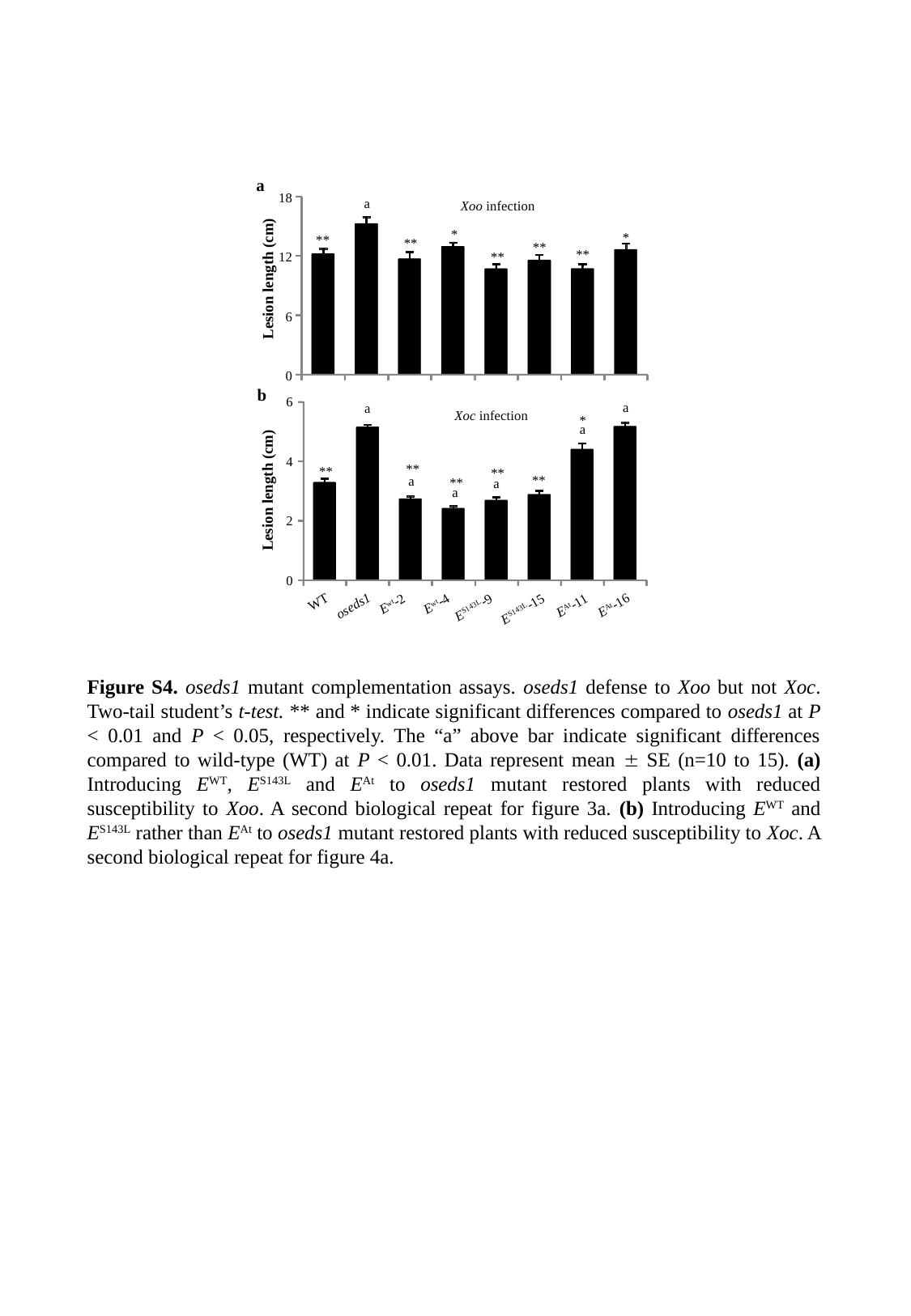

a
18
a
Xoo infection
*
*
**
**
**
**
**
12
Lesion length (cm)
6
0
b
6
a
a
*
Xoc infection
a
**
**
**
**
**
a
a
a
4
Lesion length (cm)
2
0
WT
Ewt-2
Ewt-4
EAt-11
EAt-16
oseds1
ES143L-9
ES143L-15
Figure S4. oseds1 mutant complementation assays. oseds1 defense to Xoo but not Xoc. Two-tail student’s t-test. ** and * indicate significant differences compared to oseds1 at P < 0.01 and P < 0.05, respectively. The “a” above bar indicate significant differences compared to wild-type (WT) at P < 0.01. Data represent mean ± SE (n=10 to 15). (a) Introducing EWT, ES143L and EAt to oseds1 mutant restored plants with reduced susceptibility to Xoo. A second biological repeat for figure 3a. (b) Introducing EWT and ES143L rather than EAt to oseds1 mutant restored plants with reduced susceptibility to Xoc. A second biological repeat for figure 4a.

## Slide 5
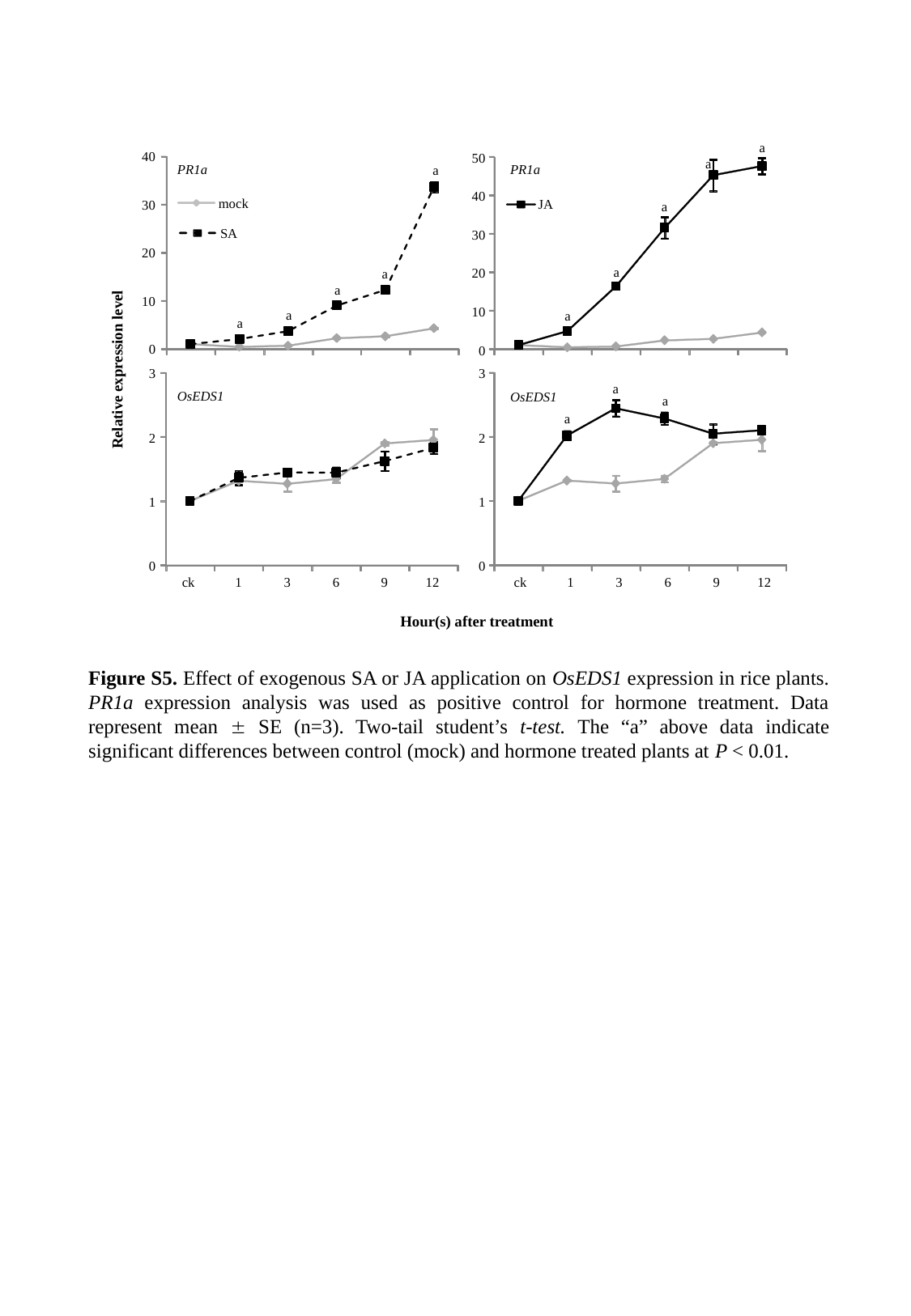

a
40
a
50
a
a
a
a
a
PR1a
PR1a
40
a
mock
JA
30
SA
30
20
a
20
10
a
10
0
0
Relative expression level
3
3
a
a
OsEDS1
OsEDS1
a
2
2
1
1
0
0
ck
1
3
6
9
12
ck
1
3
6
9
12
Hour(s) after treatment
Figure S5. Effect of exogenous SA or JA application on OsEDS1 expression in rice plants. PR1a expression analysis was used as positive control for hormone treatment. Data represent mean ± SE (n=3). Two-tail student’s t-test. The “a” above data indicate significant differences between control (mock) and hormone treated plants at P < 0.01.

## Slide 6
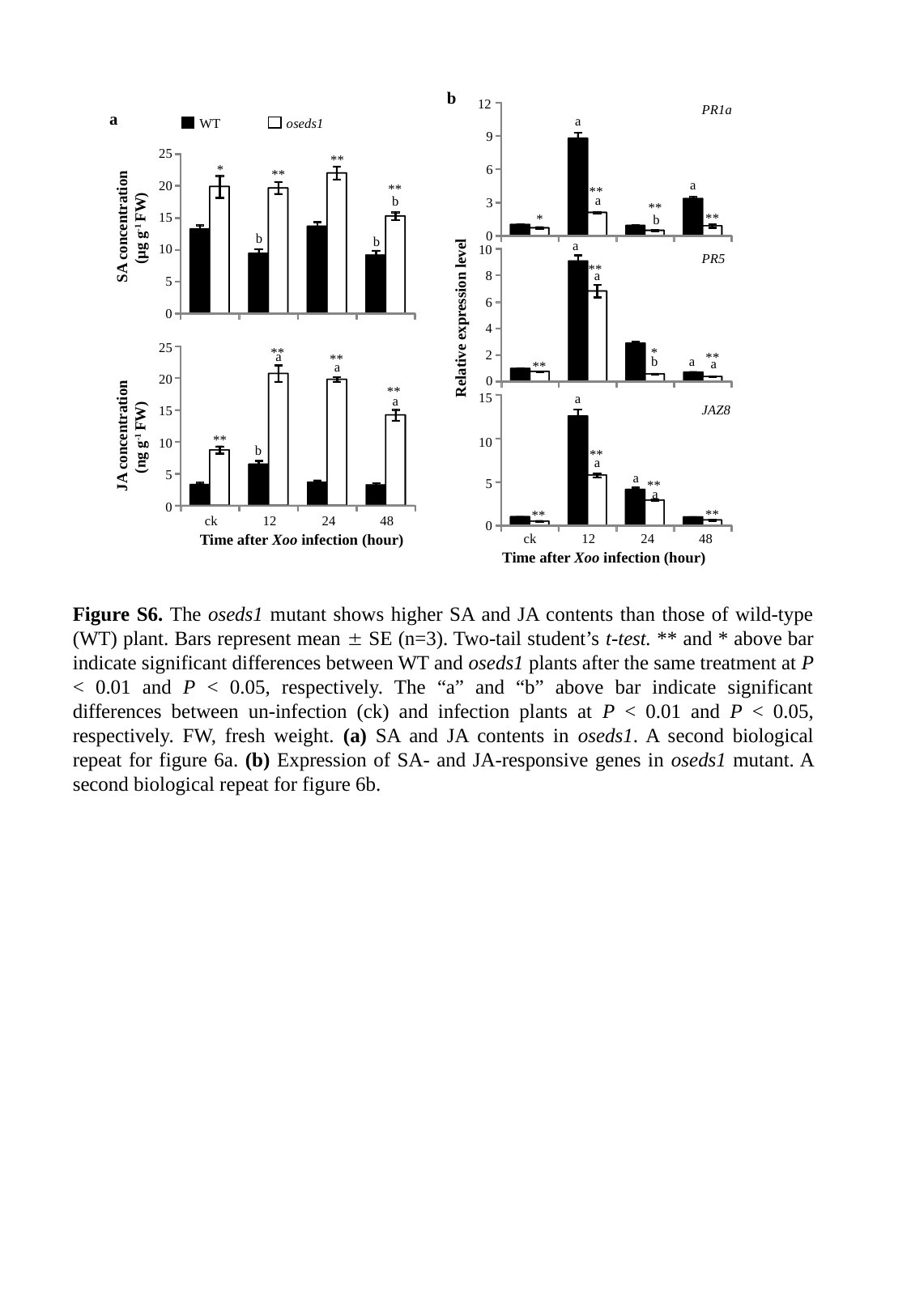

b
12
PR1a
a
a
WT
oseds1
9
25
**
*
**
6
a
**
20
**
a
b
**
3
**
 SA concentration
(µg g-1 FW)
*
b
15
b
b
0
a
10
10
PR5
**
a
8
5
6
Relative expression level
0
4
**
*
25
a
**
**
2
a
b
a
**
a
20
0
**
a
a
15
JAZ8
15
 JA concentration
(ng g-1 FW)
**
10
10
b
**
a
a
5
**
5
a
0
**
**
ck
12
24
48
0
Time after Xoo infection (hour)
ck
12
24
48
Time after Xoo infection (hour)
Figure S6. The oseds1 mutant shows higher SA and JA contents than those of wild-type (WT) plant. Bars represent mean ± SE (n=3). Two-tail student’s t-test. ** and * above bar indicate significant differences between WT and oseds1 plants after the same treatment at P < 0.01 and P < 0.05, respectively. The “a” and “b” above bar indicate significant differences between un-infection (ck) and infection plants at P < 0.01 and P < 0.05, respectively. FW, fresh weight. (a) SA and JA contents in oseds1. A second biological repeat for figure 6a. (b) Expression of SA- and JA-responsive genes in oseds1 mutant. A second biological repeat for figure 6b.

## Slide 7
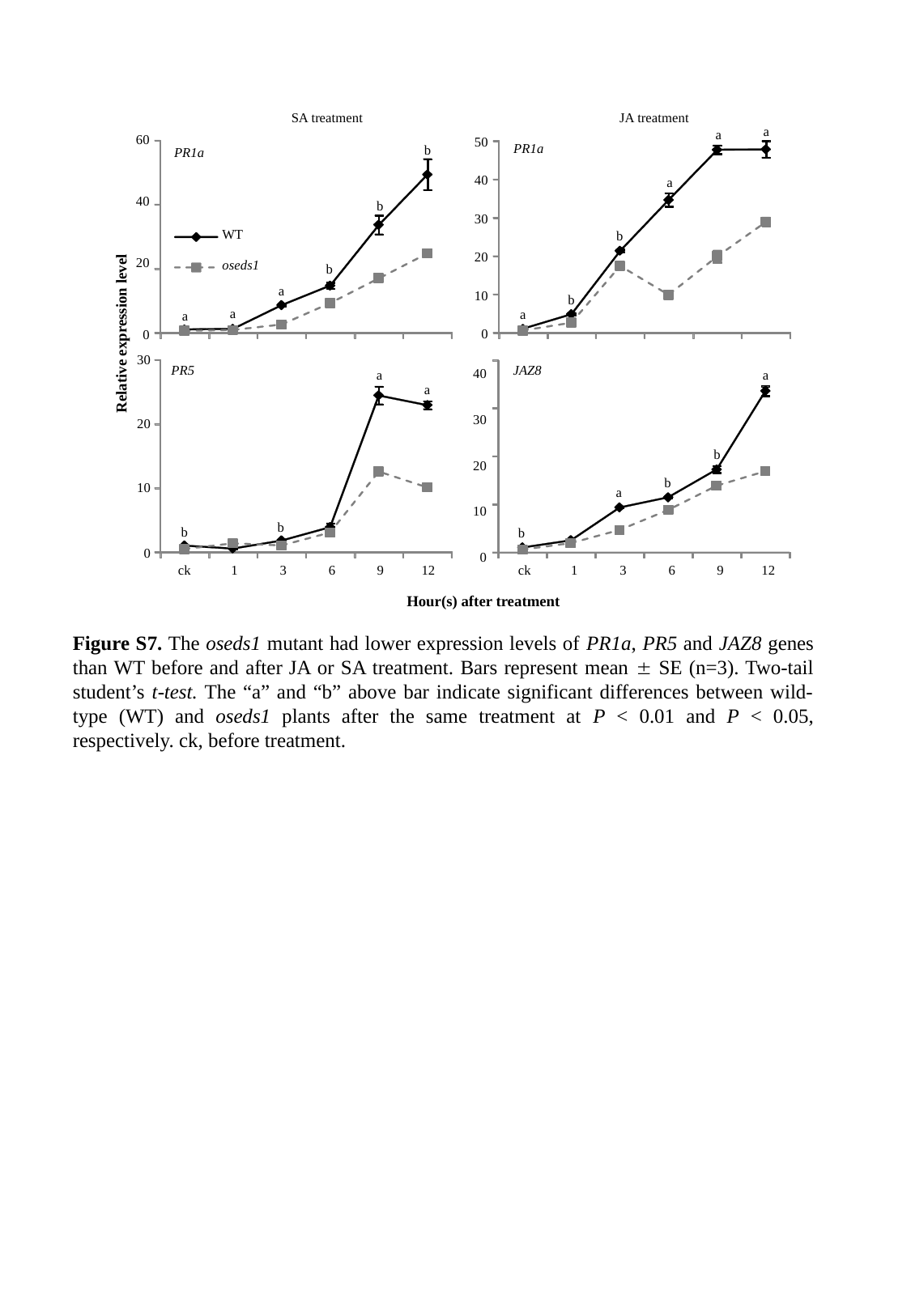

SA treatment
JA treatment
a
a
60
50
b
PR1a
PR1a
a
40
b
40
30
b
WT
20
20
b
oseds1
a
b
10
a
a
a
Relative expression level
0
0
30
a
a
PR5
JAZ8
40
a
30
20
b
20
b
a
10
10
b
b
b
0
0
ck
1
3
6
9
12
ck
1
3
6
9
12
Hour(s) after treatment
Figure S7. The oseds1 mutant had lower expression levels of PR1a, PR5 and JAZ8 genes than WT before and after JA or SA treatment. Bars represent mean ± SE (n=3). Two-tail student’s t-test. The “a” and “b” above bar indicate significant differences between wild-type (WT) and oseds1 plants after the same treatment at P < 0.01 and P < 0.05, respectively. ck, before treatment.
